# Characterisation of the immune compounds in koala milk using a combined transcriptomic and proteomic approach

**DOI:** 10.1038/srep35011

**Published:** 2016-10-07

**Authors:** Katrina M. Morris, Denis O’Meally, Thiri Zaw, Xiaomin Song, Amber Gillett, Mark P. Molloy, Adam Polkinghorne, Katherine Belov

**Affiliations:** 1 Faculty of Veterinary Science, University of Sydney, Camperdown, NSW, 2006, Australia; 2 Australian Proteome Analysis Facility, Dept. Chemistry and Biomolecular Sciences, E8C310, Research Park Drive, Macquarie University, NSW, 2109. Australia; 3 Australia Zoo Wildlife Hospital, Beerwah, Queensland 4519, Australia; 4 Centre for Animal Health Innovation, Faculty of Science, Health, Education and Engineering, University of the Sunshine Coast, Maroochydore, Qld, 4558, Australia

## Abstract

Production of milk is a key characteristic of mammals, but the features of lactation vary greatly between monotreme, marsupial and eutherian mammals. Marsupials have a short gestation followed by a long lactation period, and milk constituents vary greatly across lactation. Marsupials are born immunologically naïve and rely on their mother’s milk for immunological protection. Koalas (*Phascolarctos cinereus*) are an iconic Australian species that are increasingly threatened by disease. Here we use a mammary transcriptome, two milk proteomes and the koala genome to comprehensively characterise the protein components of koala milk across lactation, with a focus on immune constituents. The most abundant proteins were well-characterised milk proteins, including β-lactoglobulin and lactotransferrin. In the mammary transcriptome, 851 immune transcripts were expressed, including immunoglobulins and complement components. We identified many abundant antimicrobial peptides, as well as novel proteins with potential antimicrobial roles. We discovered that marsupial VELP is an ortholog of eutherian Glycam1, and likely has an antimicrobial function in milk. We also identified highly-abundant koala endogenous-retrovirus sequences, identifying a potential transmission route from mother to young. Characterising the immune components of milk is key to understanding protection of marsupial young, and the novel immune compounds identified may have applications in clinical research.

Mammals have a complex immune system that predates the divergence of eutherians and marsupials, with these two groups having broad commonalities in the gene content, structure and function of their immune systems^1^,^2^,^3^,^4^. However, a crucial difference exists in their mode of reproduction. While eutherians have a long gestation period with extended uterine development of young, marsupials have a short gestation followed by a prolonged period of development, usually in the pouch^5^. Marsupial young are born undeveloped and immunologically naïve. At birth they do not have an adaptive immune system^6^,^7^ yet are able to survive the pouch environment, known to contain an array of potential pathogenic bacterial and fungal species^8^,^9^. After an extended early lactation period during which the young is fixed to the mother’s teat, marsupial young emerge from the pouch and face new immune challenges during mid and late lactation as they are exposed to novel pathogens outside the pouch.

Due to the long period of extra-uterine development, marsupial and eutherian lactation differs in several ways. While the composition of eutherian milk is relatively consistent after an initial colostral stage^5^, the volume and composition of macronutrients, micronutrients and protein content of marsupial milk changes dramatically throughout lactation^10^,^11^,^12^,^13^. The concentration of immune compounds including immunoglobulins (Igs) varies throughout lactation to meet the changing needs of marsupial young^13^,^14^,^15^,^16^. This distinct form of lactation, where milk provides crucial immune protection of the developing young across the period of lactation^14^,^15^,^16^,^17^, is reflected in the immunogenetic repertoire of marsupials. For instance, previous research has discovered an expanded repertoire of cathelicidins in marsupials that are expressed in the mammary gland and have broad spectrum antibacterial activity including against multi-drug resistant strains^1^,^18^,^19^.

Koalas (*Phascolarctos cinereus*) are a unique and iconic Australian marsupial, and the last surviving species of the family Phascolarctidae. Koalas are born after just 34–35 days of gestation^20^. Young first emerge from the pouch at around six months to seven months, but they continue to suckle for up to one year^21^. The major cellular components of koala milk are immune cells including neutrophils and macrophages^22^. The temporal pattern of milk composition in koalas differs from that of other marsupials, indicating that koalas may have adopted a different lactation strategy from other marsupial species^11^. Koalas are of interest from an immunological perspective due to the infectious diseases they carry. Most significant among these is the transmissible and recently endogenised koala gamma-retrovirus (KoRV)^23^. This virus has been implicated in immunosuppression and immunomodulation of the host, and is associated with fatal lymphoma and leukaemia^24^. In addition koalas are particularly susceptible to *Chlamydia*, which has a significant impact on female koala fertility^25^.

The aim of this study was to investigate the protein components of koala milk. To do this we used multiple data sources including a mammary transcriptome, milk proteomes from two koalas at two different stages of lactation, and the koala genome. We focus on the immune components of the milk and analyse the proteomic differences between the two stages of lactation.

## Results

### Most abundant transcripts and peptides

**Figure 1:**
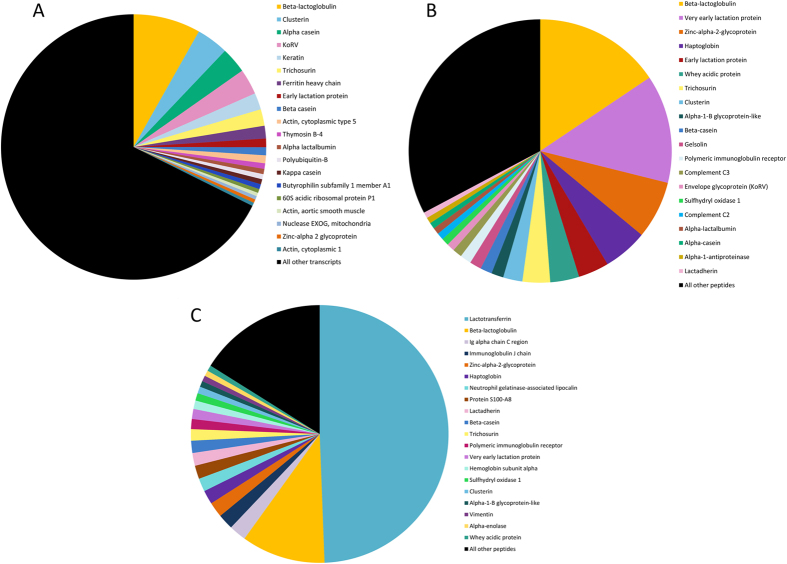
Pie charts of peptides/transcripts based on relative abundance as a percentage. Top 20 in each koala data set is labelled, with remaining peptides/transcripts grouped under ‘All other’. (**A**) Early lactation mammary gland transcriptome. (**B**) Early lactation milk proteome. (**C**) Late lactation milk proteome.

In this study we constructed a transcriptome from the mammary gland of a koala during early lactation, and two koala milk proteomes, one from early lactation and one from late lactation. The mammary transcriptome assembly contained a high number of contigs (nearly 225,000). We used CEGMA^26^ and BUSCO^27^ to search for a defined set of single-copy, conserved eukaryote and vertebrate orthologs in the assembly. CEGMA reported 245 complete alignments against the core set of 248 eukaryote orthologs (98.8% complete, up to 99.6% when partial alignments are included). BUSCO reported 85.9% complete (89.8% complete including partial alignments) of 3,023 conserved vertebrate orthologs. To remove assembly artefacts and transcriptional noise, we filtered transcripts with a Fragments Per Kilobase Million (FPKM) <1, retaining 26,852 transcripts. Of these, 10,700 transcripts have unique BLAST^28^ hits to the SwissProt non-redundant database, providing an estimate of the number of proteins encoded in the transcriptome. The early lactation proteome includes 230 peptides, while the late lactation proteome identified 235 peptides. As the milk samples were snap frozen prior to our analyses we cannot exclude that the origin of some proteins detected in koala milk proteome result from lysis of cells in the milk. The two proteomes shared 106 peptides in common (46.1%). The 50 most highly expressed transcripts in the mammary gland transcriptome and the 50 most abundant peptides in the milk proteomes are shown in Table 1, while pie charts showing the percent abundance of the top 20 transcripts/peptides are shown in Fig. 1. See Supplementary Tables S1–3 for the top 200 expressed transcripts in the transcriptome and all transcripts matched in the proteomes.

Known major milk proteins were identified among the most abundant transcripts and peptides. β-lactoglobulin, a major nutrient protein^29^, was the most abundant transcript and peptide in the early lactation sample and was the second most abundant peptide in the late lactation sample. All three caseins (α, β and κ), key nutritional compounds in the milk, were highly abundant in all the samples investigated, and beta casein was the most abundant casein in both early and late lactation samples (10^th^ most abundant peptide in both). Trichosurin, a protein unique to marsupials, was highly abundant in both early and late lactation (7^th^ and 11^th^ most abundant peptide respectively). In the late lactation milk sample, lactotransferrin was the most highly abundant peptide and was far more abundant (24.47% of peptides) than in the early lactation milk sample (0.08% of peptides). Late lactation protein (LLP), which is highly expressed during late lactation in wallaby and possum^13^,^30^, was not seen in the late lactation milk proteome. LLP was expressed in the early lactation transcriptome, although at a very low abundance. It may be that our single, opportunistic, late-lactation sample missed the window of high expression of this protein in the koala. Early lactation protein (ELP) and whey acidic protein (WAP) were both highly abundant in the early lactation proteome (5^th^ and 6^th^ respectively). The second most abundant peptide in early lactation was very early lactation protein (VELP).

### Very early lactation protein

VELP is a protein previously identified in the milk of brushtail possum (*Trichosurus vulpecula*) and wallaby (*Macropus eugenii*)^31^,^32^. Homologs of this protein outside marsupials have not been previously identified. In the present study, we identified a full length koala *VELP* transcript and this was used to search the koala genome, wallaby mammary transcriptome^12^ and the Tasmanian devil (*Sarcophilus harrisii*) genome and milk transcriptome^33^, and full length orthologs were identified in these species. No ortholog could be identified in opossum (*Monodelphis domestica*) through BLAST and HMMer searches. The koala and devil sequences were identified on short scaffolds with no annotated genes, and in the devil no prediction was made at this locus by NCBI or Ensembl pipelines.

Using the *VELP* sequence we performed BLAST searches on the other available lactation transcriptome: the mammary transcriptomes from the wallaby^12^ and milk transcriptome from the devil^33^. We found that *VELP* was expressed during pregnancy, early lactation and mid-lactation in the tammar mammary gland, but not in late lactation. In the devil mid-lactation milk transcriptome, *VELP* was present but had very low expression. Koala *VELP* was very highly abundant in the early lactation milk proteome being the second most abundant peptide (13.3% of peptides). However it was also abundant in the late lactation sample being the 13th most abundant peptide.

**Figure 2:**
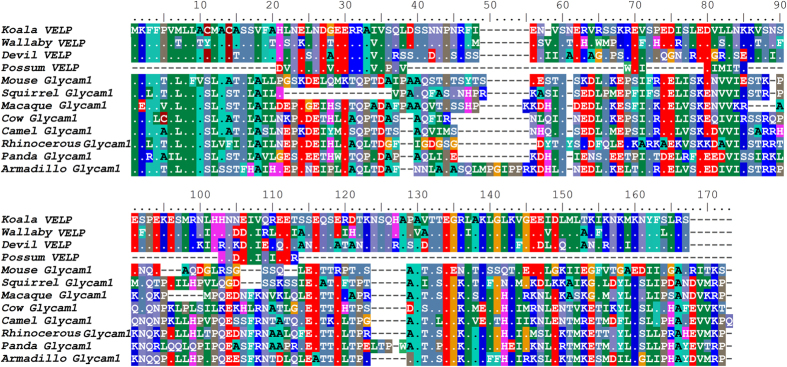
Alignment of marsupial VELP and eutherian Glycam1 (PP3/lactophorin) sequences. Dots indicate identity to the koala VELP sequence. Accession numbers: Tammar wallaby (*Macropus eugenii*; EX207743), Brushtail possum (*Trichosurus vulpecula*; P85093), Tasmanian devil (*Sarcophilus harrisii*; GEDN01008364), Mouse (*Mus musculus*; Q02596), Squirrel (*Ictidomys tridecemlineatus*; I3N1S9), Macaque (*Macaca mulata*; F7H8B2), Cow (*Bos Taurus*; P80195), Camel (*Camelus bactrianus*; P15522), Rhinocerous (*Ceratotherium simum*; XP_004429511.1), Panda (*Ailuropoda melanoleuca*; G1L2P5), Armadillo (*Dasypus novemcinctus*; ENSDNOT00000038407).

**Figure 3:**
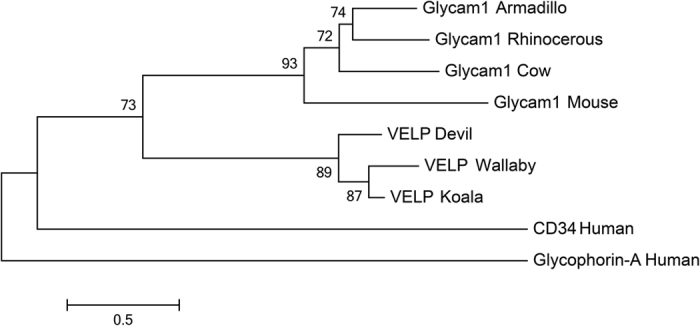
Phylogenetic tree of marsupial VELP and eutherian Glycam1 (PP3/lactophorin) sequences. Human CD34 (P28906) and Glycophorin-A (P02724) are used as outgroups. The tree was constructed using the maximum likelihood approach and the JTT model with bootstrap support values from 500 bootstrap tests. Bootstrap values less than 50% are not displayed. Accession numbers as for Fig. 2.

**Figure 4:**
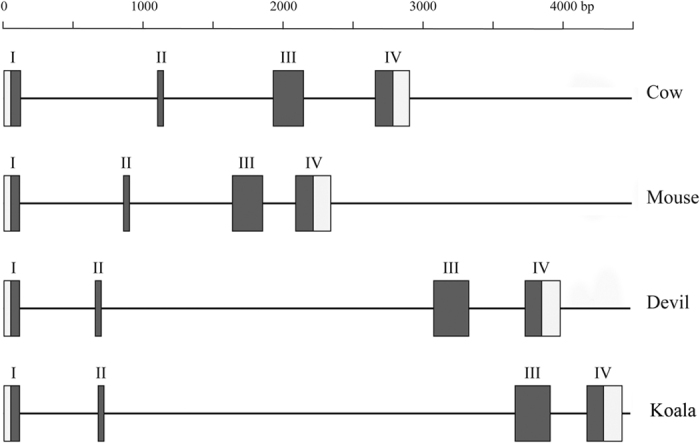
Schematic comparison of the gene structure of mouse (ENSMUSG0000002249) and cow (ENSBTAG00000013417) *Glycam1* (*PP3*) and Tasmanian devil (GEDN01008364.1) and koala *VELP*. Roman numerals indicate exon number. Filled in boxes indicate coding sequence, empty boxes indicate UTR sequences.

Using BLAST and HMMer searches with full-length marsupial *VELP* sequences, we identified homology with the eutherian protein Glycam1, also known as PP3 or lactophorin. *Glycam1* is a functional gene in only some eutherian mammals; in some species, including humans, pseudogenes have been identified^34^. To evaluate the relationship between VELP and Glycam1 further, we constructed an alignment using all available VELP and Glycam1 protein sequences (Fig. 2) and maximum-likelihood phylogenetic tree using all marsupial VELP sequences and a phylogenetically representative subset of eutherian Glycam1 sequences (Fig. 3). Eutherian Glycam1 sequences range from 141 to 164 residues. The marsupial sequences are similar with 159 or 160 residues. The tree groups the marsupial VELP and eutherian Gycam1 together, albeit with moderate bootstrap support (75%). We compared the genomic structure of cow and mouse *Glycam1* and devil and koala *VELP* (Fig. 4) and found that they are highly similar. The genes each have four exons and the lengths of the exons and UTRs are very similar between the four species. The divergence of these sequences is high, both within marsupials and between marsupials and eutherians. Peptide sequence identity of the koala sequence with the wallaby and devil sequences was 75% and 67.5% respectively. Identity with eutherian sequences varied between 19.3–23.4%. Comparison of the genomic context of marsupial *VELP* and eutherian *Glycam1* was not possible, because no other flanking genes are present on the relatively short devil and koala genomic scaffolds to which *VELP* maps.

### Novel milk proteins and transcripts

A novel peptide previously identified only as a transcript in the devil milk transcriptome and wallaby mammary transcriptomes (previously named *Novel Gene 1*^33^) was identified in koala milk. In the koala, this peptide was the 26^th^ and 42^nd^ most abundant peptide in the early and late lactation milk proteomes respectively (Table 1; “Marsupial Milk 1”). This peptide was not identified in any other marsupial transcriptome through BLAST searches. Homology with other proteins was not identified through BLAST or HMMER searches of the opossum genome and the SwissProt database, nor through protein domain searches. This short protein is only 98–99 residues long, and is highly divergent, with 69.6% and 66.6% sequence identity of the koala sequence with the wallaby and devil sequences respectively. In the koala genome this gene is located approximately 8 kb upstream of the *TESPA1* (Thymocyte Expressed, Positive Selection Associated 1) gene and flanked a gene that showed homology with eutherian lacritin (*LACRT*) and dermicidin (*DCD*). The orthologous region in humans encodes the genes *DCD* and *LACRT*, as well as the *GLYCAM1* pseudogene and Mucin-like 1 (*MUCL1*), each of which encode short glycoprotein peptides with antimicrobial functions.

Two putative non-coding RNA (ncRNA) transcripts were among the top 200 most highly-expressed transcripts in the mammary transcriptome (see Supplementary Table S1). One was the 39^th^ most highly-expressed transcript, which showed homology to a devil sequence previously characterised as a ncRNA in the automated annotation of the devil genome (Ensembl release 83). A second putative ncRNA (78^th^ most highly-expressed) shares homology with a transcript expressed in the wallaby mammary transcriptome, suggesting a common function in marsupial lactation. This sequence was located approximately 1kb downstream of the *WBSCR27* gene, in both devil and opossum genomes. *WBSCR27* encodes WBS27, a methyltransferase protein. We consider this to be putatively non-coding due to the lack of homology with any known protein, absence of an open-reading frame, absence of known protein domains in Pfam, lack of introns, and absence in the koala milk proteomes.

### KoRV

In the early lactation mammary transcriptome, KoRV sequences collectively represented the fourth most highly expressed transcripts and 3% of all transcripts in the transcriptome. In the milk from the same animal the three major KoRV proteins (gag, env and pol) were collectively the 14^th^ most abundant peptides in the early milk proteome, representing 1.07% of peptides in the milk. The proportion of KoRV retroviral peptides was similar in the late lactation milk proteomes where they represented 0.73% of all peptides.

### Immune Proteins

Immune proteins were a focus in this study due to the important role milk plays in immune defence of marsupial young. By performing a BLAST search of the early lactation mammary transcriptome with the Immune Database for Monotremes and Marsupials (IDMM^35^), 851 genes with primary immune function present in the mammary transcriptome were identified (see Supplementary Table S4). This represents approximately 9% of all genes expressed in the koala mammary gland. Among these are lysozyme, cathelicidins, immunoglobulins, complement factors, cytokines, and MHC I and II. The 50 most highly expressed immune transcripts are shown in Table 2. The top three proteins have roles in both nutrient transport and immune defence. These are ferritin, which aids in iron transfer but also sequesters free iron to prevent bacterial growth^36^, zinc-alpha 2 glycoprotein which is involved in lipid mobilisation and immunoregulation^37^, and butyrophilin which is involved in the synthesis of milk fat globules and also has a role in immune regulation^38^.

### Immunoglobulins and Ig receptors

Igs in the milk have a crucial role in protecting marsupial young. IgG and IgA are the main isotypes of Igs present in eutherian and marsupial milk^14^,^39^,^40^,^41^. IgG is absorbed across the gut epithelium and enters the circulatory system of the pouch young protecting against systemic infection; IgA is not absorbed but has a critical role in protecting the neonatal gut^42^,^43^. Like IgA, IgM is not absorbed, but has a role in protecting the gut. All marsupial Ig heavy and light chains were present in the koala mammary transcriptome with the exception of *IgE* (Table 3). IgA and both of the Ig light chains were more abundant in the late milk sample. IgA was highly abundant in late lactation being the third most abundant peptide. IgA had a large difference in abundance between the two samples, with the IgA heavy chain comprising over 2% of peptides in the late lactation proteome, but only 0.08% of peptides in the early lactation proteome. IgG had a similar abundance across the two samples (0.68% and 0.46% of peptides in the early and late lactation proteomes respectively). IgM was more abundant in early lactation where it comprised 0.25% of all peptides, but it was not detected in the late milk sample.

Polymeric Immunoglobulin Receptor (PIgR), involved in transfer and protection of IgA and IgM^44^, was highly abundant in both early and late lactation being the 12^th^ most abundant protein in both proteomes. FcRN, a component of the Fc receptor which allows for the transport of IgG across the intestinal wall of the neonate^45^, was only identified in early lactation (228^th^ protein).

### Alpha 1B glycoprotein-like

**Figure 5:**
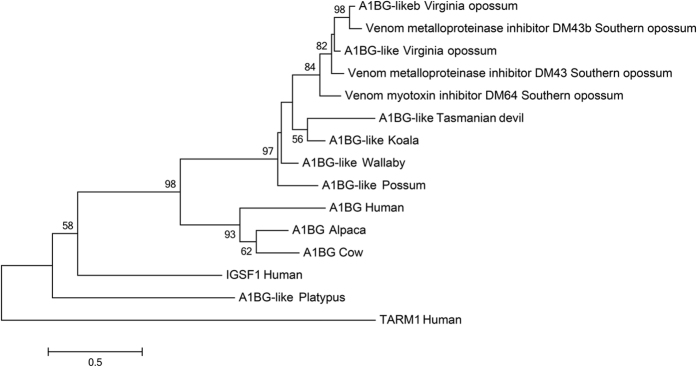
Phylogenetic tree of eutherian A1BG, opossum DM43 and DM46, and A1BG-like sequences in marsupials. Human TARM1 and IGSF1, related members of the immunoglobulin superfamily are used as outgroups. The tree was constructed using the maximum likelihood approach and the JTT model with bootstrap support values from 500 bootstrap tests. Bootstrap values less than 50% are not displayed. Accession numbers: Tasmanian devil (*Sarcophilus harrisii;* XP_012402143), Wallaby (*Macropus eugenii;* FY619507), Possum (*Trichosurus vulpecula*; DY596639) Virginia opossum (*Didelphis virginiana*; AAA30970, AAN06914), Southern opossum (*Didelphis marsupialis*; AAL82794, P82957, AAN64698), Human (*Homo sapiens*; P04217, B6A8C7, Q8N6C5), Platypus (*Ornithorhychus anatinus*; ENSOANP00000000762), Cow (*Bos taurus*; Q2KJF1), Alpaca (*Vicugna pacos*; XP_015107031).

A peptide identified in the late and early milk proteomes showed homology to eutherian alpha 1B glycoprotein (A1BG), a plasma protein with unknown function^46^, as well as venom inhibitors characterised in the Southern opossum *Didelphis marsupialis* (DM43 and DM46^47^,^48^,^49^), all members of the immunoglobulin superfamily. To characterise the relationship between the peptide sequence identified in koala, A1BG, DM43 and DM46, a phylogenetic tree was constructed (Fig. 5) including all marsupial and monotreme homologs (identified by BLAST), three phylogenetically representative eutherian sequences, with human IGSF1 and TARM1, related members of the immunoglobulin super family, used as outgroups. This phylogeny indicates that A1BG-like proteins in marsupials and the *Didelphis* antitoxic proteins are homologs of eutherian A1BG, with excellent bootstrap support (98%). The marsupial A1BG-like sequences and the *Didelphis* antitoxic proteins formed a single clade with strong bootstrap support (97%).

The wallaby and devil A1BG-like sequences were not present in the devil milk transcriptome^33^ and the tammar mammary transcriptome^12^ (determined by BLAST). However, this protein was highly abundant in the koala milk, being the 9^th^ and 17^th^ most abundant peptide in the early and late lactation milk proteomes respectively, and was also in the mammary transcriptome, though at a lower abundance (0.3 × 10^−3^ of transcripts). In addition, we searched all available marsupial transcriptomes (including transcriptomes from devil, wallaby and koala) using BLAST and found that this gene was expressed in a variety of koala tissues (spleen, lymph node, liver, bone marrow, heart and brain), but was not in any other marsupial transcriptome. This protein has not been previously identified in mammalian milk and its function is unknown. The expression of this protein in many tissues across multiple individuals without evidence of acute exposure to venom does not support a role in response to envenomation in koalas. Presence in a variety of immune tissues and its homology with immunoglobulins suggests it may have an immune role. Alternatively, as it was expressed koala liver, but not the immune tissues of devil and wallaby, a role in detoxification of dietary plant compounds is also possible.

### Antimicrobial peptides (AMPs)

We examined the expression of AMPs in koala as these proteins in the milk of placental mammals have been demonstrated to prevent bacterial or viral infection. Cathelicidins have the ability to directly lyse pathogenic cells^50^, and are thought to be crucial in protecting immunologically naïve marsupial young^18^. Cathelicidins identified in the milk of the wallaby are known to have broad spectrum antibacterial activity^19^. Previously, five cathelicidins have been identified in the tammar mammary transcriptome^12^ and four in the devil milk transcriptome^33^. We found evidence of four cathelicidins in koala mammary tissue and milk. All four were expressed in the koala early lactation mammary transcriptome. One of these was detected in the early lactation milk proteome while two were detected in the late lactation milk proteomes. Cathelicidins were relatively abundant in the late lactation milk sample where they together comprised 1.1% of peptides. Several other peptides with direct antimicrobial activity were also identified. WAP four-disulfide core domain protein 2 (WFDC2) was abundant in the early lactation proteome (31^st^ most abundant protein) and was also present in the late lactation proteome (113^th^ most abundant protein). This protein has antibacterial activity against many pathogenic species of bacteria, while not showing activity against common gut commensal species^51^. Thus it is thought to protect against pathogenic species without disturbing the balance of commensal gut flora^51^. Lysozyme plays an important role in innate immunity as it is capable of lysing bacterial cells^52^, providing crucial protection to mammalian young. This protein identified in the late lactation proteome (51^st^ most abundant protein), but it was not detected in the early milk proteome, and only had a very low expression in the mammary transcriptome (0.01% of transcripts). A homolog of C10orf99 (also known as AP-57 and CSBF), was identified in the late lactation proteome (70^th^ protein). C10orf99 is an AMP that was only recently described in humans^53^,^54^. This protein has broad spectrum antimicrobial activity against gram-positive bacteria, fungal species, mycoplasma and lentivirus^54^. Although not in the top 100 most abundant peptides, peptidoglycan recognition protein 1 (PGLYRP1) and mucin-1 were also identified in the late lactation proteome. PGLYRP1 is an antibacterial protein that has a different structure and mechanism of action to other known mammalian AMPs. In humans, this protein is produced by epithelial cells, body secretions and leukocytes^55^. It is bactericidal against various pathogenic gram-positive bacteria, but not against commensal bacterial flora^55^. Additionally, it has a bacteriostatic effect against both gram-positive and gram-negative bacterial species^55^. This dual activity is thought to remove pathogenic species while limiting overgrowth of commensal species^55^. Mucin-1 can inhibit binding and invasion of bacterial pathogens in the gut^56^,^57^,^58^ and has anti-viral activity^59^.

### Complement factors

Numerous complement factors were observed to be highly abundant in both milk proteomes. Classical complement genes have been identified in the colostrum of cows^60^, and have demonstrated bactericidal activity^61^,^62^,^63^. These components also provide protection to immunoglobulins in solution^60^. In the koala mammary transcriptome, eleven complement transcripts were identified while six were present in the early milk proteome and four in the late milk proteome. This included three that were highly abundant in both proteomes, (C2, C3 and C4A), all components of the classical complement pathway. These three components were more abundant in the early milk proteome together comprising 2.41% of peptides in the proteome.

## Discussion

In order to comprehensively analyse the proteins and transcripts present in koala milk, with a specific focus on immune proteins, we have constructed a transcriptome from the mammary gland in early lactation, and two milk proteomes from early and late lactation. The most abundant koala milk proteins were similar to those of wallaby, possum and Tasmanian devil, including β-lactoglobulin, caseins, Trichosurin and α-lactalbumin^12^,^32^,^34^,^64^. Trichosurin, a protein unique to marsupials, was highly abundant in both early and late lactation. Its function is unknown, but a role in priming the neonate liver to produce detoxifying enzymes has been suggested^65^. Such a role might explain its particularly high abundance in koala milk, as their diet of eucalypt leaves is rich in phytotoxins. An ortholog of eutherian A1BG was highly abundant in both early and late milk samples, but has not previously been reported in the milk of any species. This protein may have an immune or detoxification function in koala milk.

An intriguing finding was that KoRV transcripts and peptides were highly abundant in all samples (3% of transcripts, ~1% of peptides). This indicates that retroviral particles are being transmitted in the mother’s milk. In primates, transmission of GALV, the closely related gibbon ape leukaemia virus, occurs *in utero*, postnatally, via contact and via faeces^66^. In koalas, vertical transmission of the exogenous form of KoRV does not occur via the germ line^67^. Previous studies have suggested that KoRV may be transmitted *in utero* or in the milk^67^. Should the milk-borne viral particles prove infectious, we provide the first evidence for a vertical transmission route via the mother’s milk.

Identification of a full-length *VELP* transcript allowed us to demonstrate that this gene is orthologous to *Glycam1* (*PP3*). The expression of both VELP and Glycam1 in milk^68^,^69^,^70^, their sequence similarity and common gene structure provides strong evidence that marsupial *VELP* is orthologous to eutherian *Glycam1*. This protein was very highly abundant in the early lactation milk, comprising 13.3% of peptides. In contrast to wallaby and possum^31^,^32^, this protein was also abundant in koala late lactation. The divergence of these sequences is high, comparable to some of the most divergent immune proteins^4^. In cows, antibacterial activity of this protein against Gram positive and negative bacteria^71^,^72^, and antiviral activity^73^ has been demonstrated. It is therefore highly likely that VELP has antibacterial activity and may be key for protection of marsupial young.

A novel protein was the 26^th^ most abundant protein in early lactation. Previously only the transcript encoding this protein had been identified in devil milk and wallaby mammary transcriptomes^33^, where it was referred to as *Novel Gene 1*. Homology to any protein could not be found through BLAST and HMMer searches. As this peptide appears to be unique to marsupials and has lactation-specific expression, we propose calling this gene and protein Marsupial Milk 1 (*MM1*). Interestingly, koala *MM1* was located in a region that shares synteny with a genomic region in eutherian mammals that encodes several proteins with antimicrobial functions, including Glycam1, lacritin, dermcidin and mucin-like 1^74^,^75^. Each of these proteins have a glandular expression pattern, including mammary, lacrimal and sweat glands^74^,^75^. Although no clear homology was identified between *MM1* and these genes, we propose that its genomic context and similar glandular expression suggest a relationship to this group of genes, and that therefore it may have a similar antimicrobial function.

Several proteins were much more abundant in the early lactation sample than in the late lactation sample, most notably ELP, WAP, WFDC2, and VELP. Each of these proteins have potential immune roles; while ELP and WAP are both thought to prevent degradation of Igs in the gut^76^,^77^, WFDC2 and VELP likely play antimicrobial roles. Lactotransferrin, a protein key for iron transport and which aids in immune defence^78^, was much more abundant in the late lactation sample. Additional immune proteins, including Igs, complement and AMPs also showed a difference in expression between the two milk proteomes. These immune peptides may have a stage-dependent function during lactation, perhaps reflecting different microbial exposure at different stages.

In general Igs, in particular IgA, were more abundant in the late lactation proteome. This matches observations in possum, wallaby and common wollaroo (*Macropus robustus*), where IgA is highly upregulated from mid-lactation^14^,^39^,^40^. The elevated abundance of IgA in late lactation may aid in protecting the young from novel pathogens as it emerges from the pouch and shifts to a solid diet. Abundance of IgG was approximately the same in the early and late lactation proteomes; however FcRN, crucial for IgG transport across the gut epithelium, was only detected in early lactation. IgM was only detected in early lactation; this Ig may be important for protecting the gut of koala young while IgA expression in milk is low.

Three classical complement components (C2, C3 and C4A), were highly abundant, particularly in the early proteome (2.4% of peptides). To our knowledge, this is the first time classical complement components have been reported in marsupial milk. As classical complement factors have demonstrated bactericidal activity^61^,^62^,^63^ and provide protection to Igs in solution^60^, they may have a key role in protection of koala young, particularly in early lactation.

A large range of AMPs was identified in the koala lactation samples. Studies in placental mammals have demonstrated that AMPs in the milk can prevent bacterial or viral infection^57^,^59^; thus AMPs acting in concert are likely to have a crucial role in protecting the gut of the young from pathogens. WFDC2, a protein that protects against pathogenic species without disturbing the balance of commensal gut flora^52^, was highly abundant in koala early lactation. Mucin-1, which inhibits binding and invasion of pathogens in the gut^57^,^58^, was also detected in early lactation.

AMPs abundant in late lactation included cathelicidins, lysozyme, PGLYRP1, and C10orf99. Four cathelicidins, proteins thought to be crucial in protecting marsupial young^18^, were identified. Lysozyme, which is expressed in the milk of a range of eutherian and marsupial species^32^,^33^,^64^,^79^, was highly abundant in the late lactation proteome. C10orf99, a newly identified broad-spectrum antimicrobial, has previously only been identified in human, where it is expressed in the mucosa of the gastrointestinal tract^54^, but has not been reported in milk. PGLYRP1 is an antibacterial protein that has a dual activity, removing pathogenic species while limiting overgrowth of commensal species^55^. This protein could be crucial during late lactation, protecting against pathogens and regulating gut flora as the koala young develops its own gut flora to aid in digestion of solid food.

Currently there is a major research focus on identifying and testing antimicrobial peptides for clinical purposes as an alternative to conventional antibiotics. Most previous research has been on eutherian mammal AMPs. Cathelicidins expressed in wallaby milk have been previously found to be highly potent against pathogenic bacteria, including multi-drug resistant species^18^. Koala cathelicidins identified in this study can now be tested to see if they have similar potency. In addition to cathelicidins, many other AMPs were identified, some of which have not been previously reported in the milk of any mammalian species. These would make excellent targets for further investigation into the antimicrobial function with a long term aim for clinical application. Furthermore, a number novel proteins were identified in this study, and future research should focus on identifying their function. Synthesising and assaying these proteins will enable us to determine what role they play in the protection of pouch young. The findings of this study will also have applications for the process of hand-raising koala young, which is an important step in the conservation of koalas, particularly in areas of decline. Through increasing our understanding of the composition of koala milk at different stages of lactation, milk substitutes provided to hand-raised koala young may be improved in the future.

## Conclusions

Koalas, like other marsupials, are born underdeveloped and grow through an extended period of lactation with distinct developmental stages. They are highly reliant on the mother’s milk to protect them from pathogens in the pouch, and from novel pathogens encountered as they emerge from the pouch. In this study we have provided an insight into the protein components of koala milk. Due to the difficulty of obtaining such samples this study is limited to two time points and two individuals, and future studies may aim to extend this to a greater number of individuals at different stages as samples become available. The proteins found in koala milk showed many commonalities with marsupial milk investigated in previous studies, although some notable differences occurred. This includes the high abundance of trichosurin and A1BG, potentially linked to the specialised diet of the koala, and the very high abundance of VELP which likely has an antimicrobial role. We have identified a range of immune proteins critical for protection of the underdeveloped koala young, both prior to, and after emergence from the pouch. This contributes to our understanding of how marsupial young are able to survive and provides us with a host of distinct immune proteins that can be investigated for their function and clinical potential.

## Methods

### Ethics Statement

As samples were collected from animals at necropsy following euthanasia as part of their routine veterinary care, their collection was considered by the University of the Sunshine Coast Animal Ethics Committee and considered exempt from requiring further approval (AN/E/15/06).

### Milk and mammary sample collection

Samples were collected at necropsy from two female koalas admitted to Australia Zoo Wildlife Hospital for veterinary care. The koala “Leah” was euthanized following identification of an osteochondroma while the koala “Little Jo” was euthanized due to severe dog attack injuries (Table 4). Little Jo was in the early stage of lactation with an attached pouch young of 18 g, aged between 2 and 10 weeks old. Both milk and mammary gland tissue were collected from Little Jo. Milk was collected from Leah who was in the late stage of lactation with a young aged 8 months. The mammary gland sample was stored in RNA-later and the milk samples were snap frozen.

### Koala milk sample preparation for proteomics

The frozen milk samples were thawed and 100 μL was diluted in 400 μL of ultrapure water and spun at 14,000 ×g for one hour to separate the top lipid layer from the water soluble fraction (containing whey and casein). From the water soluble fraction, 100 μL was taken and spun in pre-conditioned centrifugal filter unit (10,000Da MWCO, Millipore) at 13,000 × g for 10 minutes at 10 °C. The retained fraction was collected and 20 μL was reduced with 5 mM dithiothreitol for one hour at 56 °C, alkylated with 10 mM iodoacetamide at RT for one hour and digested overnight with trypsin at 37 °C. The digested samples were dried in a vacuum centrifuge.

### Strong Cation Exchange (SCX) High Performance Liquid Chromatography (HPLC)

The dried, digested peptide samples from both early and late lactating koala milks were independently fractionated by SCX HPLC (1260 Quaternary HPLC system with Polysulfoethyl A, 200 mm × 2.1 mm, 5 μm, 200 Å column; Agilent). The samples were resuspended in loading buffer (5 mM potassium Phosphate, 25% ACN, pH 2.7). After sample loading and washing, buffer B (5 mM potassium Phosphate, 350 mM KCl, 25% acetonitrile, pH 2.7) was increased from 10% to 45% in 70 minutes and then increased to 100% for 10 minutes at a flow rate 300 μl/min. The SCX HPLC eluent was collected every 2 minutes from the start of the gradient for 30 mins and then at 4 minute intervals for the next 40 mins. The elutes were pooled into 13 fractions, dried and used for nanoLC ESI analysis.

### High pH Reversed-Phase Peptide Fractionation

An aliquot of dried, digested early lactation milk was fractionated using a Pierce™ High pH Reversed-Phase Peptide Fractionation Kit, (Thermo Scientific). The spin column was conditioned with acetonitrile (centrifugation at 5,000 *g* for 2 min; repeated twice) followed by 0.1% trifluoroacetic acid (centrifugation at 5,000 *g* for 2 min; repeated twice). The sample was resuspended in 300 μL of 0.1% trifluoroacetic acid and 150 μL was loaded onto the column. The flow-through fraction was collected by centrifugation at 3,000 *g* for 2 min then washed by adding 300 μL of Milli-Q water and spun at 3,000 *g* for 2 min. A total of eight fractions were eluted by centrifugation at 3,000 *g* for 2 min using the eight step-high-pH elution solutions following the manufacturers specification (5% acetonitrile (ACN), 0.095% triethylamine (TEA); 7.5% ACN, 0.093% TEA; 10% ACN, 0.09% TEA; 12.5% ACN, 0.088% TEA; 15% ACN, 0.085% TEA; 17.5% ACN, 0.083% TEA; 20% ACN, 0.08% TEA; 50% ACN, 0.05% TEA). The eight fractions were dried and used for nanoLC-MS/MS analysis.

### NanoLC ESI MS/MS data acquisition

A 5600 TripleTOF mass spectrometer (AB Sciex) coupled to an Eksigent Ultra-nanoLC-1D system (Eksigent, Dublin, CA) was employed for LC-MS/MS analysis. Each of the dried peptidefractions were resuspended in 80 μL of loading/desalting solution (0.1% (v/v) formic acid, 2% (v/v) acetonitrile). Forty μL of sample was injected onto a reverse phase peptide C18 Captrap (Bruker) for pre-concentration and desalted for 5 minutes with the loading buffer at a flow rate of 10 μL per minute. After desalting, the peptide trap was switched in-line with an in-house packed analytical column (75 μm × 10 cm) directly in a fused silica PicoTip emitter (New Objective, Woburn, MA, USA) with solid core Halo C18, 160 Å, 2.7 μm (Bruker). Peptides were eluted from the column using the buffer B (99.9% (v/v) acetonitrile, 0.1% (v/v) formic acid) gradient starting from 10% and increasing to 40% over 45 minutes at a flow rate of 300 nL per minute. After peptide elution, the column was flushed with 95% buffer B for 10 minutes and re-equilibrated with 95% buffer A (0.1% (v/v) formic acid) for 12 minutes before next sample injection. The peptides were analysed in the positive ion nanoflow electrospray mode in an information dependent acquisition (IDA) mode.

TOF-MS survey scan was acquired at m/z 350-1500 with 0.25 second accumulation time, with the ten most intense precursor ions (2+−5+; counts >150) in the survey scan consecutively isolated for subsequent automated measurement of their corresponding product ions. Dynamic exclusion was used with a 20 sec exclusion time and 50 ppm precursor mass window. Product ion spectra were generated using rolling collision energy and accumulated for 200 msec over the 100–1500 m/z range.

### Data processing

The data were exported using ABSciexCommandDriver.exe (AB Sciex) in a format suitable for submission to the database search software, Mascot v2.4 Daemon (Matrix Science Ltd, London, UK). The search parameters were as follows: Variable modifications: Carbamidomethyl (C), Oxidation (M); Peptide tol. ±: 20 ppm; MS/MS tol. ±: 0.1 Da; Peptide charge: 2+−4+; Enzyme: Trypsin. A database was constructed based on transcripts obtained from the koala mammary gland (sequencing and assembly described below). A decoy database of reverse sequences was used to report 1% peptide false discovery rate (FDR). Protein abundances were estimated using the exponentially modified protein abundance index (emPAI) method^80^. The mass spectrometry proteomics data have been deposited to the ProteomeXchange Consortium via the PRIDE partner repository with the dataset identifier PXD003726.

### RNA isolation and sequencing

RNA was isolated from 100 mg mammary gland preserved in RNA-later (QIAGEN) using TRI-Reagent (Sigma-Aldrich) following the manufacturer’s instructions, including the additional step to remove fat. This was followed by DNA removal using DNase I (Sigma Aldrich). The RNA quantity and quality was checked on a Nano-Drop 2000 (Thermo Scientific) and on a Bioanalyser (Agilent Technologies). The final yield of total RNA was approximately 3 μg and the RNA integrity number was 7.1. Library construction and sequencing were performed by The Ramaciotti Centre (UNSW, Kensington, NSW) with TruSeq chemistry on a HiSeq2000 (Illumina). The 100 bp paired end reads were submitted to the NCBI Sequence Read Archive (BioProject [PRJNA327021], and BioSample [SAMN05300458]).

### Transcriptome assembly and annotation

RNAseq reads were assembled with the Trinity pipeline (version 2.0.6)^81^ using the default parameters and the options -trimmomatic and -normalize reads. This assembly resulted in 224,496 contigs, with a mean length of 1,213 bp, and a transcript sum of 272 Mb. The longest scaffold was 24, 321 bp and the shortest 224 bp. We checked the assembly for completeness against single-copy, conserved eukaryotic genes using the core set in CEGMA and the vertebrate set in BUSCO, using the transcriptome option. Functional annotation of the koala milk transcriptome was performed using the Trinotate pipeline (version 2.0.2) (https://trinotate.github.io/). In brief, BLASTp was performed using koala mammary predicted ORFs as the query and the SwissProt non-redundant database (provided with Trinotate) as the target and the de novo transcripts aligned against the same using tBLASTx. HMMER and Pfam databases^82^,^83^ were used to predict protein domains, SignalP (version 4.1^84^) to predict the presence of signal peptides, RNAmmer (version 2.3.2^85^) to predict ribosomal DNA, and TMHMM (version 2^86^) to predict transmembrane helices within the predicted ORFs from the milk transcriptome. These transcriptome annotations were loaded into an SQLite database, and abundance estimation was performed using the RSEM method^87^.

The number of protein coding genes was estimated by determining the number of unique SwissProt entries identified through the BLASTp search. A list of immune transcripts in the mammary transcriptome was generated by searching the milk transcriptome with proteins from the Immunome Database for Marsupials and Monotremes (IDMM)^35^ using tBLASTn. IDMM is a manually curated database of immune genes obtained from a number of marsupials including the Tasmanian devil, tammar wallaby, brushtail possum, northern brown bandicoot (*Isoodon macrourus*) and opossum and the monotremes platypus (*Ornithorhynchus anatinus*) and echidna (*Tachyglossus aculeatus*).

The top 200 transcripts in the mammary transcriptome, all transcripts matched in the proteome and all transcripts in the immune list were manually checked to confirm identity using the following methods. Transcripts with no BLAST hits or poor quality BLAST hits (E value > 10^−12^) were checked through additional BLAST searches to the NCBI database, marsupial transcriptomes or to marsupial genomes on Ensembl. For those transcripts whose identity could still not be confidently assigned through these methods, further searches and analyses were performed. Searches to the Pfam and Rfam databases were used to identify conserved protein or RNA domains. For genes where marsupial homologs were identified, including *VELP* and *MM1*, HMM profiles were constructed and used to search, using HMMsearch^88^, additional marsupial genomes and the SwissProt database. For genes where potential homologs were identified, including *VELP*, *MM1*, and *AB1G-like*, alignments and phylogenetic trees were constructed to examine the relationships between the genes. Alignments were produced in BioEdit^89^ using ClustalW alignment^90^. For phylogenetic tree construction, protein sequences were aligned using the MUSCLE algorithm^91^ in MEGA6^92^, with default parameters. Phylogenetic trees were constructed using the maximum-likelihood method and the Jones-Thornton-Taylor (JTT) model^93^, and evaluation through 500 bootstrap replicates in MEGA6. The exon structure of devil and koala *VELP* was examined by identifying the scaffolds encoding this gene through TBLASTN to the respective genome, then using the *VELP* protein sequences and the identified contigs as inputs to FGENESH+^94^. The identity of several very short transcripts could not be conclusively determined due to the short length and these have been assigned as Unknown.

### Data Availability

The data sets supporting the results of the article are available in the Short Read Archive repository, (Bioproject [PRJNA327021], Biosample [SAMN05300458]) and the PRIDE repository [PXD003726].

## Additional Information

**How to cite this article**: Morris, K. M. *et al*. Characterisation of the immune compounds in koala milk using a combined transcriptomic and proteomic approach. *Sci. Rep*. **6**, 35011; doi: 10.1038/srep35011 (2016).

## Supplementary Material

Supplementary Information

## Figures and Tables

**Table 1:** Top 50 most abundant transcripts/peptides in the mammary transcriptome and two milk proteomes.

Rank	Early Lactation Mammary Transcriptome	% total	Early Lactation Milk Proteome	% total	Late Lactation Milk Proteome	% total
1	Beta-lactoglobulin	8.19	Beta-lactoglobulin	15.59	Lactotransferrin	49.4
2	Clusterin	3.9	Very early lactation protein	13.3	Beta-lactoglobulin	10.53
3	Alpha Casein	3.16	Zinc-alpha-2-glycoprotein	7.1	Ig alpha chain C region	2.16
4	KoRV	3.13	Haptoglobin	5.46	Immunoglobulin J chain	1.96
5	Keratin	2.03	Early lactation protein	3.77	Zinc-alpha-2-glycoprotein	1.91
6	Trichosurin	2.02	Whey acidic protein	3.56	Haptoglobin	1.73
7	Ferritin heavy chain	1.55	Trichosurin	3.4	Neutrophil gelatinase-associated lipocalin	1.67
8	Early lactation protein	1.05	Clusterin	2.37	Protein S100-A8	1.67
9	Beta casein	0.97	Alpha-1-B glycoprotein-like	1.48	Lactadherin	1.6
10	Actin, cytoplasmic type 5	0.96	Beta-casein	1.44	Beta-casein	1.53
11	Thymosin B-4	0.68	Gelsolin	1.44	Trichosurin	1.44
12	Alpha-lactalbumin	0.65	Polymeric immunoglobulin receptor	1.39	Polymeric immunoglobulin receptor	1.27
13	Polyubiquitin-B	0.63	Complement C3	1.15	Very early lactation protein	1.25
14	Kappa casein	0.62	Envelope glycoprotein (KoRV)	0.97	Hemoglobin subunit alpha	1.07
15	Butyrophilin subfamily 1 member A1	0.6	Sulfhydryl oxidase 1	0.91	Sulfhydryl oxidase 1	0.94
16	60S acidic ribosomal protein P1	0.47	Complement C2	0.86	Clusterin	0.85
17	Actin, aortic smooth muscle	0.44	Alpha-lactalbumin	0.81	Alpha-1-B glycoprotein-like	0.74
18	Nuclease EXOG, mitochondria	0.44	Alpha-casein	0.77	Vimentin	0.74
19	Zinc-alpha 2 glycoprotein	0.43	Alpha-1-antiproteinase	0.75	Alpha-enolase	0.72
20	Actin, cytoplasmic 1	0.41	Lactadherin	0.73	Whey acidic protein	0.72
21	Insulin like growth factor	0.38	Neutrophil gelatinase-associated lipocalin	0.71	Nucleobindin-1	0.71
22	Very early lactation protein	0.36	Hemoglobin subunit beta	0.69	Peptidyl-prolyl cis-trans isomerase A	0.71
23	Marsupial Milk 1	0.36	Ig gamma chain C region	0.68	Transketolase	0.6
24	Actin, ACTM	0.36	Nucleobindin-1	0.64	Peroxiredoxin-1	0.59
25	Whey acidic protein	0.34	Hemoglobin subunit alpha	0.55	Protein S100-A9	0.59
26	Cytochrome c	0.32	Marsupial milk 1	0.54	Ig kappa chain C region	0.58
27	Transgelin	0.31	Actin, aortic smooth muscle	0.52	78 kDa glucose-regulated protein	0.56
28	Matrix gla protein	0.28	78 kDa glucose-regulated protein	0.51	Cathepsin L1	0.56
29	40S ribosomal protein S18	0.28	Lipopolysaccharide-binding protein	0.47	Ig lambda chain V region	0.51
30	Secreted Protein, Acidic, Cysteine-Rich (Osteonectin)	0.28	Serum amyloid A protein	0.46	Phosphoglycerate mutase 1	0.51
31	TCDD-inducible poly(ADP-ribose) polymerase	0.26	WAP four-disulfide core domain protein 2	0.45	Actin, cytoplasmic 2	0.5
32	Chain 5, 60s Rrna	0.25	Leucine-rich alpha-2-glycoprotein	0.43	Alpha-casein	0.47
33	60S ribosomal protein L6	0.24	Monocyte differentiation antigen CD14	0.42	Ig gamma chain C region	0.46
34	Elongation factor-1	0.23	Peroxiredoxin-1	0.41	Kappa casein	0.45
35	Myosin light Polypeptide	0.23	Complement C4-A	0.4	Alpha-lactalbumin	0.44
36	Insulin-like growth factor-binding protein 5	0.23	Alpha-2-macroglobulin	0.38	Protein disulfide-isomerase A3	0.44
37	Collagen alpha-1(I) chain-like	0.22	Peroxidasin homolog	0.38	Insulin-like growth factor-binding protein 4	0.42
38	Prostoglandin-H2	0.22	Tumor necrosis factor receptor superfamily member 6B	0.38	Envelope glycoprotein (KoRV)	0.4
39	Putative ncRNA	0.2	Amyloid beta A4 protein	0.36	Ubiquitin-60S ribosomal protein L40	0.4
40	Keratin	0.2	Elastin	0.34	Pigment epithelium-derived factor	0.39
41	60S ribosomal protein L10a	0.2	Fibronectin	0.34	Profilin-1	0.39
42	MHC class I	0.2	Cathepsin L1	0.32	Marsupial milk 1	0.37
43	40S ribosomal protein S24	0.2	C-C motif chemokine 3	0.29	Gelsolin	0.35
44	Annexin A2	0.19	Ceruloplasmin	0.29	Pro-Pol polyprotein (KoRV)	0.3
45	E3 ubiquitin	0.19	Prosaposin	0.29	Retinoic acid receptor responder protein 1	0.3
46	Ribonuclease H1	0.18	Fibroblast growth factor-binding protein 2	0.26	Xanthine dehydrogenase/oxidase	0.29
47	60S ribosomal protein L10	0.18	Histone-lysine N-methyltransferase 2C	0.25	Dipeptidase 2	0.27
48	Cysteine and glycine-rich protein 1	0.18	Ig mu chain C region	0.25	Ig heavy chain V-III region VH26	0.27
49	Prothymosin alpha	0.18	Peroxiredoxin-5, mitochondrial	0.25	Leukocyte elastase inhibitor	0.27
50	Vimentin	0.17	EGF-containing fibulin-like extracellular matrix protein 1	0.24	Glucose-6-phosphate isomerase	0.26

% total calculated as the percentage of total transcript expression/peptide abundance.

**Table 2:** Top 50 most highly expressed immune transcripts in the early lactation mammary transcriptome.

Rank	Gene	Gene Name	% total
1	FTH1	Ferritin heavy chain	1.55
2	BTN1A1	Butyrophilin subfamily 1 member A1	0.60
3	AZGP1	Zinc-alpha 2 glycoprotein	0.43
4	MHCI	MHC I	0.20
5	FTL	Ferritin light chain	0.14
6	TPT1	Translationally controlled tumour protein	0.12
7	MUC1	Mucin 1	0.11
8	CD74	CD74	0.10
9	B2M	Beta 2 microglobulin	0.10
10	CD63	CD63	0.10
11	PIGR	Polymeric immunoglobulin receptor	0.09
12	MHCI	MHC I	0.08
13	CCL25	CCL chemokine 25	0.08
14	CD9	CD9	0.08
15	PRDX1	Peroxiredoxin-1	0.08
16	Phci7	Cathelicidin Phci7	0.07
17	MHCI	MHC I	0.06
18	C1QA	Complement C1q subcomponent subunit A	0.06
19	MHCII-DAB	MHC II-DAB	0.06
20	MHCII-DAA	MHC II-DAA	0.05
21	C1S	Complement C1s subcomponent	0.05
22	CTGF	Connective tissue growth factor	0.05
23	Phci1	Cathelicidin Phci1	0.05
24	IFITM2	Interferon induced transmembrane protein 2	0.04
25	SERPING1	Serpin peptidase inhibitor, clade G (C1 Inhibitor), member 1	0.04
26	THBS1	Thrombospondin 1	0.04
27	HSPA1L	Heat shock 70 kDa protein 1L	0.04
28	C1QC	Complement component 1, q subcomponent, C chain	0.04
29	ANXA5	Annexin A5	0.03
30	CALR	Calreticulin	0.03
31	EPCAM	Epithelial cell adhesion molecule	0.03
32	CLEC3B	Tetranectin	0.03
33	CCLK4	C-C motif chemokine	0.03
34	CD47	CD47	0.03
35	ACKR4	Atypical chemokine receptor 4	0.03
36	APLP2	Amyloid beta (A4) precursor-like protein 2	0.03
37	BCAP31	B-cell receptor-associated protein 31	0.03
38	MIF	Macrophage migration inhibitory factor	0.03
39	HM13	Minor histocompatibility antigen H13	0.03
40	PRDX2	Peroxiredoxin 2	0.03
41	ITGB1	Integrin, beta 1	0.03
42	LGALS1	Galectin-1	0.03
43	DAP1	Death-associated protein 1	0.03
44	THY1	Thy-1 cell surface antigen	0.03
45	PSMD8	26S proteasome non-ATPase regulatory subunit 8	0.02
46	FCGR2	Low affinity immunoglobulin gamma Fc region receptor II-b	0.02
47	MME	Neprilysin	0.02
48	IL25	Interleukin 25	0.02
49	IL1RL2	Interleukin-1 receptor-like 2	0.02
50	IL10RB	IL-10 receptor subunit beta	0.02

% total calculated as the percentage of total transcript expression.

**Table 3:** Abundance of Ig heavy and light chains in the early and late lactation proteomes.

Ig chain	EL Mammary transcriptome %	EL Milk proteome %	LL Milk Proteome %
IgA	5.93 × 10^−4^	0.08	2.16
IgG	1.43 × 10^−3^	0.68	0.46
IgE	Not detected	Not detected	Not detected
IgM	5.50 × 10^−4^	0.25	Not detected
IgK	3.98 × 10^−3^	0.10	0.58
IgL	2.75 × 10^−4^	0.11	0.51

Percentage of the total transcripts/peptides detected in the mammary transcriptome and milk proteomes. EL = early lactation, LL = late lactation.

**Table 4:** Koala sample details.

Koala	Leah	Little Jo
Samples collected	Milk	Milk and mammary gland
Weight of pouch young (g)	895	18
Age of pouch young	~8 months	Between 2 week and 10 weeks
Stage of lactation	Late lactation	Early lactation
Reason for euthanasia of mother	Osteochondroma	Dog attack
Location	Gold Coast, Qld	Somerset, Qld

## References

[b1] Belov K. . Characterization of the opossum immune genome provides insights into the evolution of the mammalian immune system. Genome Res. 17, 982–991 (2007). 17495011 10.1101/gr.6121807 PMC1899125

[b2] Morris K., Wong E. & Belov K. Use of genomic information to gain insights into immune function in marsupials: A review of divergent immune genes In Marsupial genetics and genomics (eds Deakin J. E., Waters P. D. & Graves J. A. M.) Ch. 18 (Springer, 2010).

[b3] Belov K., Miller R. D., Old J. M. & Young L. J. Marsupial immunology bounding ahead. Aust. J. Zool. 61, 24–40 (2013).

[b4] Morris K. M., Cheng Y., Warren W., Papenfuss A. T. & Belov K. Identification and analysis of divergent immune gene families within the Tasmanian devil genome. BMC Genomics 16, 1017 (2015). 26611146 10.1186/s12864-015-2206-9 PMC4662006

[b5] Lefèvre C. M., Sharp J. A. & Nicholas K. R. Evolution of lactation: ancient origin and extreme adaptations of the lactation system. Annu. Rev. Genomics Hum. Genet. 11, 219–238 (2010). 20565255 10.1146/annurev-genom-082509-141806

[b6] Old J. M. & Deane E. M. The detection of mature T- and B-cells during development of the lymphoid tissues of the tammar wallaby (*Macropus eugenii*). J. Anat. 203, 123–131 (2003). 12892411 10.1046/j.1469-7580.2003.00207.x PMC1571143

[b7] Old J. M., Selwood L. & Deane E. M. The appearance and distribution of mature T and B cells in the developing immune tissues of the stripe-faced dunnart (*Sminthopsis macroura*). J. Anat. 205, 25–33 (2004). 15255959 10.1111/j.0021-8782.2004.00310.x PMC1571326

[b8] Deakin J. E. & Cooper D. W. Characterisation of and immunity to the aerobic bacteria found in the pouch of the brushtail possum Trichosurus vulpecula. Comp. Immunol. Microbiol. Infect. Dis. 27, 33–46 (2004). 14656540 10.1016/S0147-9571(03)00013-4

[b9] Chhour K., Hinds L. A., Jacques N. A. & Deane E. M. An observational study of the microbiome of the maternal pouch and saliva of the tammar wallaby, *Macropus eugenii*, and of the gastrointestinal tract of the pouch young. Microbiol. 156, 798–808 (2010). 10.1099/mic.0.031997-0 19833775

[b10] Jolly S. E., Morriss G. A., Scobie S. & Cowan P. E. Composition of milk of the common brushtail possum, *Trichosurus vulpecula* (Marsupialia: Phalangeridae): Concentrations of elements. Aust. J. Zool. 44, 479–486 (1996).

[b11] Krockenberger A. K. Composition of the milk of the koala, Phascolarctos cinereus, an arboreal folivore. Physiol. Zool. 69, 701–718 (1996).

[b12] Lefèvre C. M., Digby M. R., Whitley J. C., Strahm Y. & Nicholas K. R. Lactation transcriptomics in the Australian marsupial, *Macropus eugenii*: transcript sequencing and quantification. BMC Genomics 8, 417 (2007). 17997866 10.1186/1471-2164-8-417 PMC2204018

[b13] Joss J. L., Molloy M. P., Hinds L. & Deane E. A longitudinal study of the protein components of marsupial milk from birth to weaning in the tammar wallaby (*Macropus eugenii*). Dev. Comp. Immunol. 33, 152–161 (2009). 18778730 10.1016/j.dci.2008.08.002

[b14] Adamski F. M. & Demmer J. Immunological protection of the vulnerable marsupial pouch young: two periods of immune transfer during lactation in *Trichosurus vulpecula* (brushtail possum). Dev. Comp. Immunol. 24, 491–502 (2000). 10785274 10.1016/s0145-305x(00)00012-4

[b15] Adamski F. M. & Demmer J. Two stages of increased IgA transfer during lactation in the marsupial, *Trichosurus vulpecula* (brushtail possum). J. Immunol. 162, 6009–6015 (1999). 10229840

[b16] Daly K. A. . Analysis of the expression of immunoglobulins throughout lactation suggests two periods of immune transfer in the tammar wallaby (*Macropus eugenii*). Vet. Immunol. Immunopathol. 120, 187–200 (2007). 17727962 10.1016/j.vetimm.2007.07.008

[b17] Edwards M. J., Hinds L. A., Deane E. M. & Deakin J. E. A review of complementary mechanisms which protect the developing marsupial pouch young. Dev. Comp. Immunol. 37, 213–220 (2012). 22504164 10.1016/j.dci.2012.03.013

[b18] Wang J. . Ancient antimicrobial peptides kill antibiotic-resistant pathogens: Australian mammals provide new options. PLoS One 6, e24030 (2011). 21912615 10.1371/journal.pone.0024030 PMC3166071

[b19] Wanyonyi S. S., Sharp J. A., Khalil E., Lefevre C. & Nicholas K. R. Tammar wallaby mammary cathelicidins are differentially expressed during lactation and exhibit antimicrobial and cell proliferative activity. Comp. Biochem. Physiol. A Mol. Integr. Physiol. 160, 431–439 (2011). 21824524 10.1016/j.cbpa.2011.07.015

[b20] Johnston S. D., McGowan M. R., O’Callaghan P. O., Cox R. & Nicolson V. Studies on the oestrous cycle, oestrus and pregnancy in the koala (*Phascolarctos cinereus*). J. Reprod. Fertil. 120, 49–57 (2000). 11006145 10.1530/jrf.0.1200049

[b21] Martin R. & Handasyde K. The koala: natural history, conservation and management 2nd edn, (Krieger Pub Co, 1999).

[b22] Young L. J. & Deane E. M. Cellular composition of the late milk of the koala (*Phascolarctos cinereus*). Aust. J. Zool. 49, 195–202 (2001).

[b23] Simmons G. S. . Prevalence of koala retrovirus in geographically diverse populations in Australia. Aust. Vet. J. 90, 404–409 (2012). 23004234 10.1111/j.1751-0813.2012.00964.x

[b24] Denner J. & Young P. R. Koala retroviruses: characterization and impact on the life of koalas. Retrovirology 10, 108 (2013). 24148555 10.1186/1742-4690-10-108 PMC4016316

[b25] Polkinghorne A., Hanger J. & Timms P. Recent advances in understanding the biology, epidemiology and control of chlamydial infections in koalas. Vet. Microbiol. 165, 214–223 (2013). 23523170 10.1016/j.vetmic.2013.02.026

[b26] Parra G., Bradnam K. & Korf I. CEGMA: a pipeline to accurately annotate core genes in eukaryotic genomes. Bioinformatics 23, 1061–1067 (2007). 17332020 10.1093/bioinformatics/btm071

[b27] Simão F. A., Waterhouse R. M., Ioannidis P., Kriventseva E. V. & Zdobnov E. M. BUSCO: assessing genome assembly and annotation completeness with single-copy orthologs. Bioinformatics 31, 3210–3212 (2015). 26059717 10.1093/bioinformatics/btv351

[b28] Altschul S. F., Gish W., Miller W., Myers E. W. & Lipman D. J. Basic local alignment search tool. J. Mol. Biol. 215, 403–410 (1990). 2231712 10.1016/S0022-2836(05)80360-2

[b29] Sawyer L. B-lactoglobulin In Advanced dairy chemistry – I. Proteins. Part A. (eds Fox P. F., McSweeney P.) 319–386 (Kluwer Academic, 2003).

[b30] Brennan A. J. . The tammar wallaby and fur seal: models to examine local control of lactation. J. Dairy Sci. 90 Suppl 1, E66–75 (2007). 17517753 10.3168/jds.2006-483

[b31] Kuy S., Kelly V. C., Smit A. M., Palmer D. J. & Cooper G. J. Proteomic analysis of whey and casein proteins in early milk from the marsupial *Trichosurus vulpecula*, the common brushtail possum. Comp. Biochem. Physiol. Part D Genomics Proteomics 2, 112–120 (2007). 20483284 10.1016/j.cbd.2007.01.002

[b32] Joss J., Molloy M., Hinds L. & Deane E. Proteomic analysis of early lactation milk of the tammar wallaby (*Macropus eugenii*). Comp. Biochem. Physiol. Part D Genomics Proteomics 2, 150–164 (2007). 20483289 10.1016/j.cbd.2007.02.002

[b33] Hewavisenti R. V. . The identification of immune genes in the milk transcriptome of the Tasmanian devil (*Sarcophilus harrisii*). PeerJ 4, e1569 (2016). 26793426 10.7717/peerj.1569 PMC4715465

[b34] Rasmussen L. K., Johnsen L. B., Petersen T. E. & Sørensen E. S. Human GlyCAM-1 mRNA is expressed in the mammary gland as splicing variants and encodes various aberrant truncated proteins. Immunol. Lett. 83, 73–75 (2002). 12057858 10.1016/s0165-2478(02)00084-6

[b35] Wong E. S. W., Papenfuss A. T. & Belov K. Immunome database for marsupials and monotremes. BMC Immunol. 12, 48 (2011). 21854560 10.1186/1471-2172-12-48 PMC3173380

[b36] Brock J. H. Lactoferrin in human milk: its role in iron absorption and protection against enteric infection in the newborn infant. Arch. Dis. Child. 55, 417–421 (1980). 7002055 10.1136/adc.55.6.417 PMC1626933

[b37] Hassan M. I., Waheed A., Yadav S., Singh T. P. & Ahmad F. Zinc alpha 2-glycoprotein: a multidisciplinary protein. Mol. Cancer. Res. 6, 892–906 (2008). 18567794 10.1158/1541-7786.MCR-07-2195

[b38] Smith I. A. . BTN1A1, the mammary gland butyrophilin, and BTN2A2 are both inhibitors of T cell activation. J. Immunol. 184, 3514–3525 (2010). 20208008 10.4049/jimmunol.0900416

[b39] Deane E. M., Cooper D. W. & Renfree M. B. Immunoglobulin G levels in fetal and newborn tammar wallabies (*Macropus eugenii*). Reprod. Fertil. Dev. 2, 369–375 (1990). 2120744 10.1071/rd9900369

[b40] Deane E. M. & Cooper D. W. Immunology of pouch young marsupials. I levels of immunoglobulin, transferrin and albumin in the blood and milk of euros and wallaroos (hill kangaroos: *Macropus robustus*, Marsupialia). Dev. Comp. Immunol. 8, 863–867 (1984). 6519341 10.1016/0145-305x(84)90069-7

[b41] Hurley W. L. & Theil P. K. Perspectives on immunoglobulins in colostrum and milk. Nutrients 3, 442–474 (2011). 22254105 10.3390/nu3040442 PMC3257684

[b42] Yadav M. The transmissions of antibodies across gut of pouch-young marsupials. Immunol. 21, 839–851 (1971). PMC1408160 5115612

[b43] Woof J. M. & Kerr M. A. The function of immunoglobulin A in immunity. J. Pathol. 208, 270–278 (2006). 16362985 10.1002/path.1877

[b44] Kaetzel C. S. The polymeric immunoglobulin receptor: bridging innate and adaptive immune responses at mucosal surfaces. Immunol. Rev. 206, 83–99 (2005). 16048543 10.1111/j.0105-2896.2005.00278.x

[b45] Jones E. A. & Waldman T. A. The mechanism of intestinal uptake and transcellular transport of IgG in the neonatal rat. J. Clin. Invest. 51, 2916–2927 (1972). 5080417 10.1172/JCI107116 PMC292442

[b46] Ishioka N., Takahashi N. & Putnam F. W. Amino acid sequence of human plasma alpha 1B-glycoprotein: homology to the immunoglobulin supergene family. Proc. Natl. Acad. Sci. USA 83, 2363–2367 (1986). 3458201 10.1073/pnas.83.8.2363 PMC323297

[b47] Neves-Ferreira A. G., Cardinale N., Rocha S. L., Perales J. & Domont G. B. Isolation and characterization of DM40 and DM43, two snake venom metalloproteinase inhibitors from *Didelphis marsupialis* serum. Biochim. Biophys. Acta. 1474, 309–320 (2000). 10779682 10.1016/s0304-4165(00)00022-2

[b48] Neves-Ferreira A. G. . Structural and functional analyses of DM43, a snake venom metalloproteinase inhibitor from *Didelphis marsupialis* serum. J. Biol. Chem. 277, 13129–13137 (2002). 11815628 10.1074/jbc.M200589200

[b49] Rocha S. L. . Functional analysis of DM64, an antimyotoxic protein with immunoglobulin-like structure from *Didelphis marsupialis* serum. Eur. J. Biochem. 269, 6052–6062 (2002). 12473101 10.1046/j.1432-1033.2002.03308.x

[b50] Bals R. & Wilson J. M. Cathelicidins–a family of multifunctional antimicrobial peptides. Cell. Mol. Life Sci. 60, 711–720 (2003). 12785718 10.1007/s00018-003-2186-9 PMC11138611

[b51] Watt A. P., Sharp J. A., Lefevre C. & Nicholas K. R. WFDC2 is differentially expressed in the mammary gland of the tammar wallaby and provides immune protection to the mammary gland and the developing pouch young. Dev. Comp. Immunol. 36, 584–590 (2012). 22024352 10.1016/j.dci.2011.10.001

[b52] Jollès P. & Jollès J. What’s new in lysozyme research? Always a model system, today as yesterday. Mol. Cell. Biochem. 63, 165–189 (1984). 6387440 10.1007/BF00285225

[b53] Pan W. . CSBF/C10orf99, a novel potential cytokine, inhibits colon cancer cell growth through inducing G1 arrest. Sci. Rep. 4, 6812 (2014). 25351403 10.1038/srep06812 PMC4212244

[b54] Yang M. . AP-57/C10orf99 is a new type of multifunctional antimicrobial peptide. Biochem. Biophys. Res. Commun. 457, 347–352 (2015). 25585381 10.1016/j.bbrc.2014.12.115

[b55] Lu X. . Peptidoglycan recognition proteins are a new class of human bactericidal proteins. J. Biol. Chem. 281, 5895–5907 (2006). 16354652 10.1074/jbc.M511631200

[b56] Schroten H. . Inhibition of adhesion of S-fimbriated Escherichia coli to buccal epithelial cells by human milk fat globule membrane components: a novel aspect of the protective function of mucins in the nonimmunoglobulin fraction. Infect. Immun. 60, 2893–2899 (1992). 1377184 10.1128/iai.60.7.2893-2899.1992 PMC257251

[b57] Schroten H. . Inhibition of adhesion of S-fimbriated Escherichia coli to epithelial cells by meconium and feces of breast-fed and formula-fed newborns: mucins are the major inhibitory component. J. Pediatr. Gastroenterol. Nutr. 15, 150–158 (1992). 1357127 10.1097/00005176-199208000-00009

[b58] Liu B., Yu Z., Chen C., Kling D. E. & Newburg D. S. Human milk mucin 1 and mucin 4 inhibit Salmonella enterica serovar Typhimurium invasion of human intestinal epithelial cells *in vitro*. J. Nutr. 142, 1504–1509 (2012). 22718031 10.3945/jn.111.155614 PMC3397338

[b59] Habte H. H. . Antiviral activity of purified human breast milk mucin. Neonatology 92, 96–104 (2007). 17361093 10.1159/000100808

[b60] Korhonen H., Marnila P. & Gill H. S. Milk immunoglobulins and complement factors. Br. J. Nutr. 84, S75–80 (2000). 11242450 10.1017/s0007114500002282

[b61] Brock J. H., Ortega F. & Pineiro A. Bactericidal and haemolytic activity of complement in bovine colostrum and serum: effect of proteolytic enzymes and ethylene glycol tetraacetic acid (EGTA). Ann. Immunol. (Paris) 126C, 439–451 (1975). 813560

[b62] Eckblad W. P., Hendrix K. M. & Olson D. P. Total complement hemolytic activity of colostral whey and sera from dairy cows. Cornell Vet. 71, 54–58 (1981). 7226847

[b63] Korhonen H. . Bactericidal effect of bovine normal and immune serum, colostrum and milk against Helicobacter pylori. J. Appl. Bacteriol. 78, 655–656 (1995). 7615421 10.1111/j.1365-2672.1995.tb03112.x

[b64] Demmer J., Ross I. K., Ginger M. R., Piotte C. K. & Grigor M. R. Differential expression of milk protein genes during lactation in the common brushtail possum (*Trichosurus vulpecula*). J. Mol. Endocrinol. 20, 37–44 (1998). 9513080 10.1677/jme.0.0200037

[b65] Watson R. P., Demmer J., Baker E. N. & Arcus V. L. Three-dimensional structure and ligand binding properties of trichosurin, a metatherian lipocalin from the milk whey of the common brushtail possum *Trichosurus vulpecula*. Biochem. J. 408, 29–38 (2007). 17685895 10.1042/BJ20070567 PMC2049081

[b66] Kawakami T. G., Sun L. & McDowell T. S. Natural transmission of gibbon leukemia virus. J. Natl. Cancer Inst. 61, 1113–1115 (1978). 212567

[b67] Xu W. . An exogenous retrovirus isolated from koalas with malignant neoplasias in a US zoo. Proc. Natl. Acad. Sci. USA 110, 11547–11552 (2013). 23798387 10.1073/pnas.1304704110 PMC3710800

[b68] Groenen M. A., Dijkhof R. J. & van der Poel J. J. Characterization of a GlyCAM1-like gene (glycosylation-dependent cell adhesion molecule 1) which is highly and specifically expressed in the lactating bovine mammary gland. Gene 158, 189–195 (1995). 7607540 10.1016/0378-1119(95)00138-v

[b69] Le Provost F., Cassy S., Hayes H. & Martin P. Structure and expression of goat GLYCAM1 gene: lactogenic-dependent expression in ruminant mammary gland and interspecies conservation of the proximal promoter. Gene 313, 83–89 (2003). 12957379 10.1016/s0378-1119(03)00632-2

[b70] Kappeler S., Farah Z. & Puhan Z. Alternative splicing of lactophorin mRNA from lactating mammary gland of the camel (*Camelus dromedarius*). J. Dairy Sci. 82, 2084–2093 (1999). 10531593 10.3168/jds.S0022-0302(99)75450-0

[b71] Campagna S., Mathot A. G., Fleury Y., Girardet J. M. & Gaillard J. L. Antibacterial activity of lactophoricin, a synthetic 23-residues peptide derived from the sequence of bovine milk component-3 of proteose peptone. J. Dairy Sci. 87, 1621–1626 (2004). 15453475 10.3168/jds.S0022-0302(04)73316-0

[b72] Barzyk W. S., Campagna S., Więcław K., Korchowiec B. & Rogalska E. The affinity of two antimicrobial peptides derived from bovine milk proteins for model lipid membranes. Colloids Surf. 343, 104–110 (2009).

[b73] Inagaki M. . The bovine lactophorin C-terminal fragment and PAS6/7 were both potent in the inhibition of human rotavirus replication in cultured epithelial cells and the prevention of experimental gastroenteritis. Biosci. Biotechnol. Biochem. 74, 1386–1390 (2010). 20622446 10.1271/bbb.100060

[b74] McKown R. L. . A cleavage-potentiated fragment of tear lacritin is bactericidal. J. Biol. Chem. 289, 22172–22182 (2014). 24942736 10.1074/jbc.M114.570143 PMC4139230

[b75] Schittek B. . Dermcidin: a novel human antibiotic peptide secreted by sweat glands. Nat. Immunol. 2, 1133–1137 (2001). 11694882 10.1038/ni732

[b76] Simpson K., Shaw D. & Nicholas K. Developmentally-regulated expression of a putative protease inhibitor gene in the lactating mammary gland of the tammar wallaby, *Macropus eugenii*. Comp. Biochem. Physiol. B Biochem. Mol. Biol. 120, 535–541 (1998). 9787813 10.1016/s0305-0491(98)10040-8

[b77] Dandekar A. M., Robinson E. A., Appella E. & Qasba P. K. Complete sequence analysis of cDNA clones encoding rat whey phosphoprotein: homology to a protease inhibitor. Proc. Natl. Acad. Sci. USA 79, 3987–3991 (1982). 6955785 10.1073/pnas.79.13.3987 PMC346561

[b78] Farnaud S. & Evans R. W. Lactoferrin–a multifunctional protein with antimicrobial properties. Mol. Immunol. 40, 395–405 (2003). 14568385 10.1016/s0161-5890(03)00152-4

[b79] Piotte C. P., Marshall C. J., Hubbard M. J., Collet C. & Grigor M. R. Lysozyme and alpha-lactalbumin from the milk of a marsupial, the common brush-tailed possum (*Trichosurus vulpecula*). Biochim. Biophys. Acta 1336, 235–242 (1997). 9305795 10.1016/s0304-4165(97)00033-0

[b80] Ishihama Y. . Exponentially modified protein abundance index (emPAI) for estimation of absolute protein amount in proteomics by the number of sequenced peptides per protein. Mol. Cell. Proteomics 4, 1265–1272 (2005). 15958392 10.1074/mcp.M500061-MCP200

[b81] Haas B. J. . De novo transcript sequence reconstruction from RNA-seq using the Trinity platform for reference generation and analysis. Nat. Protoc. 8, 1494–1512 (2013). 23845962 10.1038/nprot.2013.084 PMC3875132

[b82] Finn R. D., Clements J. & Eddy S. R. HMMER web server: interactive sequence similarity searching. Nucleic Acids Res. 39, W29–W37 (2011). 21593126 10.1093/nar/gkr367 PMC3125773

[b83] Punta M. . The Pfam protein families database. Nucleic Acids Res. 40, D290–D301 (2012). 22127870 10.1093/nar/gkr1065 PMC3245129

[b84] Petersen T. N., Brunak S., von Heijne G. & Nielsen H. SignalP 4.0: discriminating signal peptides from transmembrane regions. Nat. Methods. 8, 785–786 (2011). 21959131 10.1038/nmeth.1701

[b85] Lagesen K. . RNAmmer: consistent and rapid annotation of ribosomal RNA genes. Nucleic Acids Res. 35, 3100–3108 (2007). 17452365 10.1093/nar/gkm160 PMC1888812

[b86] Krogh A., Larsson B., von Heijne G. & Sonnhammer E. L. Predicting transmembrane protein topology with a hidden Markov model: application to complete genomes. J. Mol. Biol. 305, 567–580 (2001). 11152613 10.1006/jmbi.2000.4315

[b87] Li B. & Dewey C. N. RSEM: accurate transcript quantification from RNA-Seq data with or without a reference genome. BMC Bioinformatics 12, 323 (2011). 21816040 10.1186/1471-2105-12-323 PMC3163565

[b88] Eddy S. R. Profile hidden Markov models. Bioinformatics 14, 755–763 (1998). 9918945 10.1093/bioinformatics/14.9.755

[b89] Hall T. A. BioEdit: a user-friendly biological sequence alignment editor and analysis program for Windows 95/98/NT. Nucleic Acids Symp. Ser. 41, 95–98 (1999).

[b90] Thompson J. D., Higgins D. G. & Gibson T. J. CLUSTAL W: improving the sensitivity of progressive multiple sequence alignment through sequence weighting, position-specific gap penalties and weight matrix choice. Nucleic Acids Res. 22, 4673–4680 (1994). 7984417 10.1093/nar/22.22.4673 PMC308517

[b91] Edgar R. C. MUSCLE: multiple sequence alignment with high accuracy and high throughput. Nucleic Acids Res. 32, 1792–1797 (2004). 15034147 10.1093/nar/gkh340 PMC390337

[b92] Tamura K., Stecher G., Peterson D., Filipski A. & Kumar S. MEGA6: Molecular Evolutionary Genetics Analysis Version 6.0. Mol. Biol. Evol. 30, 2725–2729 (2013). 24132122 10.1093/molbev/mst197 PMC3840312

[b93] Jones D. T., Taylor W. R. & Thornton J. M. The rapid generation of mutation data matrices from protein sequences. Comput. Appl. Biosci. 8, 275–282 (1992). 1633570 10.1093/bioinformatics/8.3.275

[b94] Solovyev V. V. Statistical approaches in Eukaryotic gene prediction In Handbook of Statistical Genetics (eds Balding D., Cannings C. & Bishop M.) (Wiley-Interscience, 2007).

