# Supplementary material for: Characterisation of the immune compounds in koala milk using a combined transcriptomic and proteomic approach
Source: Sci Rep. 2016 Oct 7;6:35011. doi: 10.1038/srep35011 (PMC5054531; doi:10.1038/srep35011)
Supplement: Supplementary Information [file srep35011-s1.pdf]

Supplementary Information

Characterisation of the immune compounds in koala milk using a combined transcriptomic and proteomic approach.

Katrina M Morris, Denis O'Meally, Thiri Zaw, Xiaomin Song, Amber Gillett, Mark P. Molloy, Adam Polkinghorne, Katherine Belov

Table S1: Top 200 most highly expressed transcripts in the early lactation mammary gland transcriptome

Early Lactation Mammary Transcriptome	% total transcript expression
B lactoglobulin	8.195
Clusterin	3.903
Alpha Casein	3.157
KORV	3.129
Keratin	2.025
Trichosurin	2.023
Ferritin heavy chain	1.554
Early Lactation Protein	1.054
Beta Casein	0.972
Actin, cytoplasmic type 5	0.961
Thymosin B-4	0.677
Alpha lactalbumin	0.646
Polyubiquitin-B	0.631
Kappa casein	0.615
Butyrophilin subfamily 1 member A1	0.601
60S acidic ribosomal protein P1	0.473
Actin, aortic smooth muscle	0.437
Nuclease EXOG, mitochondria	0.437
Zinc-alpha 2 glycoprotein	0.428
Actin, cytoplasmic 1	0.406
Insulin like growth factor	0.384
Very early lactation protein	0.363
Marsupial Milk 1	0.358
Actin, ACTM	0.357
Whey acidic protein	0.345
Cytochrome c	0.319
Transgelin	0.314
Matrix gla protein	0.279
40S ribosomal protein S18	0.279
Secreted Protein, Acidic, Cysteine-Rich (Osteonectin)	0.278
TCDD-inducible poly(ADP-ribose) polymerase	0.263
Chain 5, 60s Rrna	0.250
60S ribosomal protein L6	0.237
Elongation factor-1	0.235
Myosin light Polypeptide	0.234
Insulin-like growth factor-binding protein 5	0.232
Collagen alpha-1(I) chain-like	0.224
Prostoglandin-H2	0.219
Putative ncRNA	0.203
Keratin	0.200
60S ribosomal protein L10a	0.198
MHC class I	0.197
40S ribosomal protein S24	0.196
Annexin A2	0.187

E3 ubiquitin	0.187
Ribonuclease H1	0.185
60S ribosomal protein L10	0.184
Cysteine and glycine-rich protein 1	0.184
Prothymosin alpha	0.175
Vimentin	0.170
Interleukin-1 receptor-associated kinase 3	0.166
Polyadenylate-binding protein 1	0.165
Tubulin alpha-1B chain	0.164
40S ribosomal protein S6	0.164
40S ribosomal protein SA	0.160
60S ribosomal protein L4	0.160
60S ribosomal protein L13	0.158
60S ribosomal protein L39	0.158
Pancreatic progenitor cell differentiation and proliferation factor	0.157
Destrin	0.156
Tropomyosin alpha-4 chain	0.154
40S ribosomal protein S19	0.151
Elongation factor 2	0.151
40S ribosomal protein S3	0.150
60S ribosomal protein L23	0.147
60S ribosomal protein L21	0.145
Ferritin light chain	0.142
60S ribosomal protein L30	0.139
Collagen alpha-1(III) chain	0.132
40S ribosomal protein S15	0.132
Abhydrolase domain-containing protein 2	0.131
60S ribosomal protein L28	0.131
Transmembrane 4 L6 family member 1	0.127
60S ribosomal protein L23a	0.127
40S ribosomal protein S7	0.126
40S ribosomal protein S4	0.124
Putative 60S ribosomal protein L37a	0.123
Putative ncRNA	0.121
Histone-lysine N-methyltransferase 2C	0.121
60S ribosomal protein L3	0.120
Wingless-type MMTV integration site family, member 7A-like	0.120
40S ribosomal protein S8	0.119
Annexin A1	0.116
Translationally-controlled tumor protein homolog	0.115
40S ribosomal protein S2	0.113
Fatty acid-binding protein, adipocyte	0.113
40S ribosomal protein S3a	0.110
Elongation factor 1-gamma	0.109
60S ribosomal protein L7a	0.109
Elastin-like	0.109
40S ribosomal protein S12	0.107
Mucin 1	0.105
Protein NLRC3	0.103

Integral membrane protein 2B	0.102
60S ribosomal protein L19	0.102
Keratin, type I cytoskeletal 18	0.101
Alpha-enolase	0.101
CD74	0.101
40S ribosomal protein S21	0.100
Ubiquitin-60S ribosomal protein L40	0.099
Tropomyosin beta chain	0.099
60S ribosomal protein L14	0.098
Gamma-aminobutyric acid receptor-associated protein	0.098
Endothelin-converting enzyme 1	0.098
Store-operated calcium entry-associated regulatory factor	0.097
Beta-2-microglobulin	0.096
CD63 antigen	0.096
Fructose-bisphosphate aldolase A	0.094
40S ribosomal protein S20	0.094
60S ribosomal protein L17	0.093
60S ribosomal protein L34	0.092
UTP--glucose-1-phosphate uridylyltransferase	0.091
Amyloid beta A4 protein	0.091
Polymeric immunoglobulin receptor	0.090
Low-density lipoprotein receptor-related protein 10	0.090
60S ribosomal protein L8	0.090
Ubiquitin-60S ribosomal protein L40	0.090
40S ribosomal protein S5	0.089
Cysteine-rich protein 1	0.087
Prosaposin	0.087
40S ribosomal protein S26	0.086
40S ribosomal protein S28	0.085
Bax inhibitor 1	0.085
60S ribosomal protein L26	0.085
60S ribosomal protein L35	0.084
Tropomyosin alpha-1 chain	0.084
Chloride intracellular channel protein 1	0.084
60S ribosomal protein L36	0.083
MHC class I	0.083
Profilin-1	0.082
Peptidyl-prolyl cis-trans isomerase A	0.082
40S ribosomal protein S13	0.081
C-C motif chemokine 25	0.080
Tubulin beta-7 chain	0.080
40S ribosomal protein S16	0.079
Alpha-actinin-1	0.079
Myosin regulatory light polypeptide 9	0.079
CD24	0.079
60S ribosomal protein L29	0.079
Glyceraldehyde-3-phosphate dehydrogenase	0.078
CD9 antigen	0.077
Nuclear protein 1	0.077
60S ribosomal protein L13a	0.077

Ribonuclease inhibitor	0.077
Peroxiredoxin-1	0.075
60S acidic ribosomal protein P0	0.075
Thioredoxin-interacting protein	0.074
Gelsolin	0.074
Protein S100-A11	0.074
60S ribosomal protein L22-like 1	0.073
Claudin-4	0.072
40S ribosomal protein S10	0.072
40S ribosomal protein S23	0.071
60S ribosomal protein L35a	0.070
Unknown-short transcript	0.070
Heterogeneous nuclear ribonucleoprotein A1	0.070
Nuclease-sensitive element-binding protein 1	0.070
Calponin-1	0.070
Myeloid-associated differentiation marker	0.069
Reticulon-4	0.069
Actin-related protein 2/3 complex subunit 3	0.069
60S ribosomal protein L24	0.069
Lactadherin	0.068
60S ribosomal protein L27a	0.067
Cathelicidin Phci7	0.067
Mucin-1	0.066
MHC class I	0.064
WAP four-disulfide core domain protein 2	0.064
Complement C1q subcomponent subunit A	0.064
Tubulin beta-4B chain	0.064
Prelamin-A/C	0.064
Myosin-9	0.064
Eukaryotic initiation factor 4A-II	0.063
Selenium-binding protein 1	0.063
Nucleophosmin	0.063
CCAAT/enhancer-binding protein delta	0.063
Collagen alpha-1(I) chain	0.063
Protein S100-A6	0.063
Serpin H1	0.063
Cytochrome b reductase 1	0.063
Minor histocompatibility antigen H13	0.063
40S ribosomal protein S17	0.062
60S ribosomal protein L11	0.062
Transmembrane and coiled-coil domain-containing protein 6	0.062
ATP synthase subunit beta, mitochondrial	0.062
Coiled-coil-helix-coiled-coil-helix domain-containing protein 2	0.062
40S ribosomal protein S15a	0.061
Augurin	0.061
Glutamine synthetase	0.060
Protein NDRG2	0.060
Collagen alpha-2(I) chain	0.059
60S acidic ribosomal protein P2	0.059
Pituitary tumor-transforming gene 1 protein-interacting protein	0.059

Cytochrome c oxidase subunit 6B1	0.058
Ornithine decarboxylase antizyme 1	0.058
MHC class II DAB	0.058
14-3-3 protein theta	0.058
Claudin-1	0.058
Glutathione peroxidase 1	0.058
40S ribosomal protein S25	0.057
14-3-3 protein beta/alpha	0.057
Glutathione S-transferase omega-1	0.056
Amiloride-sensitive sodium channel subunit alpha	0.056

Table S2: Complete list of proteins identified in the early lactation milk proteome

Early Lactation Milk Proteome	% total peptides
Beta-lactoglobulin	15.59
Very early lactation protein	13.3
Zinc-alpha-2-glycoprotein	7.1
Haptoglobin	5.46
Early lactation protein	3.77
Whey acidic protein	3.56
Trichosurin	3.4
Clusterin	2.37
Alpha-1-B glycoprotein-like	1.48
Beta-casein	1.44
Gelsolin	1.44
Polymeric immunoglobulin receptor	1.39
Complement C3	1.15
Envelope glycoprotein (KoRV)	0.97
Sulfhydryl oxidase 1	0.91
Complement C2	0.86
Alpha-lactalbumin	0.81
Alpha-S1-casein	0.77
Alpha-1-antiproteinase	0.75
Lactadherin	0.73
Neutrophil gelatinase-associated lipocalin	0.71
Hemoglobin subunit beta	0.69
Ig gamma chain C region	0.68
Nucleobindin-1	0.64
Hemoglobin subunit alpha	0.55
Marsupial Milk 1	0.54
Actin, aortic smooth muscle	0.52
78 kDa glucose-regulated protein	0.51
Lipopolysaccharide-binding protein	0.47
Serum amyloid A protein	0.46
WAP four-disulfide core domain protein 2	0.45
Leucine-rich alpha-2-glycoprotein	0.43
Monocyte differentiation antigen CD14	0.42
Peroxiredoxin-1	0.41
Complement C4-A	0.4
Alpha-2-macroglobulin	0.38
Peroxidasin homolog	0.38
Tumor necrosis factor receptor superfamily member 6B	0.38
Amyloid beta A4 protein	0.36
Elastin	0.34
Fibronectin	0.34
Cathepsin L1	0.32
C-C motif chemokine 3	0.29
Ceruloplasmin	0.29
Prosaposin	0.29

Fibroblast growth factor-binding protein 2	0.26
Histone-lysine N-methyltransferase 2C	0.25
Ig mu chain C region	0.25
Peroxiredoxin-5, mitochondrial	0.25
EGF-containing fibulin-like extracellular matrix protein 1	0.24
Granulins	0.21
Alpha-enolase	0.2
Follistatin-related protein 1	0.2
Brain-specific serine protease 4	0.19
Lactotransferrin	0.19
L-amino-acid oxidase	0.19
Nidogen-1	0.19
Nucleolar protein of 40 kDa	0.19
Fibulin-2	0.18
Complement factor H	0.17
MHC class I	0.17
Receptor-type tyrosine-protein phosphatase F	0.17
Xanthine dehydrogenase/oxidase	0.17
Endothelial lipase	0.16
Immunoglobulin superfamily containing leucine-rich repeat protein	0.16
Major allergen I polypeptide chain 1-like	0.16
Nucleoside diphosphate kinase B	0.16
Protein DJ-1	0.16
Actin, cytoplasmic 2	0.15
Cadherin-1	0.15
CD9 antigen	0.15
Desmocollin-2	0.15
Dickkopf-related protein 3	0.15
Tetranectin	0.15
Beta-2-microglobulin	0.14
Cathepsin F	0.14
C-C motif chemokine 25	0.14
Macrophage migration inhibitory factor	0.14
Butyrophilin subfamily 1 member A1	0.13
Dipeptidase 2	0.13
Golgi membrane protein 1	0.13
Kappa casein	0.13
Calcium-activated chloride channel regulator 1	0.12
Glutathione peroxidase 3	0.12
Kininogen-1	0.12
Lumican	0.12
Nucleobindin-2	0.12
Protein FAM3D	0.12
Selenium-binding protein 1	0.12
Angiopoietin-related protein 4	0.11
Ig lambda chain V region	0.11
Pancreatic secretory granule membrane major glycoprotein GP2	0.11
Pigment epithelium-derived factor	0.11
Ephrin-A1	0.1
Extracellular matrix protein 1	0.1

HHIP-like protein 2	0.1
Ig kappa chain C region	0.1
Lipoprotein lipase	0.1
Pro-Pol polyprotein (KoRV)	0.1
Acid ceramidase	0.09
Calsyntenin-1	0.09
Copper transport protein ATOX1	0.09
Cystatin-C	0.09
Fibulin-1	0.09
Fructose-bisphosphate aldolase A	0.09
Papilin	0.09
Prostaglandin-H2 D-isomerase	0.09
Retinol-binding protein 4	0.09
Testican-1	0.09
Vacuolar protein sorting-associated protein 54	0.09
Catalase	0.08
Glyceraldehyde-3-phosphate dehydrogenase	0.08
Ig alpha chain C region	0.08
Matrilysin	0.08
Peptidyl-prolyl cis-trans isomerase A	0.08
Peptidyl-prolyl cis-trans isomerase B	0.08
Plasma protease C1 inhibitor	0.08
Protein disulfide-isomerase A3	0.08
Thioredoxin	0.08
Acid sphingomyelinase-like phosphodiesterase 3a	0.07
Cathelicidin Phci1	0.07
Chloride intracellular channel protein 1	0.07
Platelet glycoprotein 4	0.07
Triosephosphate isomerase	0.07
C4b-binding protein alpha chain	0.06
Complement C1s subcomponent	0.06
Creatine kinase B-type	0.06
Extracellular superoxide dismutase [Cu-Zn]	0.06
Growth-regulated alpha protein	0.06
Homeobox protein Hox-B2	0.06
Inorganic pyrophosphatase	0.06
Insulin-like growth factor-binding protein 2	0.06
Inter-alpha-trypsin inhibitor heavy chain H3	0.06
Protein FAM20A	0.06
Transforming growth factor-beta-induced protein ig-h3	0.06
UPF0538 protein C2orf76 homolog	0.06
4F2 cell-surface antigen heavy chain	0.05
Beta-1,4-galactosyltransferase 1	0.05
Cytochrome c oxidase subunit 7A2, mitochondrial	0.05
Epithelial discoidin domain-containing receptor 1	0.05
G1/S-specific cyclin-E1	0.05
Golgi apparatus protein 1	0.05
Lysosomal protein NCU-G1	0.05
Semaphorin-7A	0.05
Tubulin alpha-4A chain	0.05

WD repeat-containing and planar cell polarity effector protein fritz homolog	0.05
45 kDa calcium-binding protein	0.04
Beta-enolase	0.04
Beta-hexosaminidase subunit beta	0.04
Carboxypeptidase Q	0.04
Cathepsin D	0.04
DNA-directed RNA polymerase II subunit RPB2	0.04
Epithelial cell adhesion molecule	0.04
Extracellular serine/threonine protein kinase FAM20C	0.04
Glucose-6-phosphate isomerase	0.04
Glucosylceramidase	0.04
Histone acetyltransferase KAT8	0.04
Hypoxia up-regulated protein 1	0.04
Kunitz-type protease inhibitor 1	0.04
Multiple coagulation factor deficiency protein 2 homolog	0.04
Protein FAM20A	0.04
Transmembrane protein 200C	0.04
Unknown	0.04
V-type proton ATPase subunit S1	0.04
10 kDa heat shock protein, mitochondrial	0.03
72 kDa type IV collagenase	0.03
Agrin	0.03
Beta-galactosidase	0.03
Calumenin	0.03
CD81 antigen	0.03
CMP-N-acetylneuraminate-beta-galactosamide-alpha-2,3-sialyltransferase 1	0.03
Collagen alpha-1(XII) chain	0.03
Drebrin-like protein	0.03
Elongation factor 1-alpha 1	0.03
Glycerophosphodiester phosphodiesterase domain-containing protein 3	0.03
G-protein coupled receptor 64	0.03
Hephaestin-like protein 1	0.03
Insulin-like growth factor-binding protein 4	0.03
Insulin-like growth factor-binding protein 5	0.03
Multiple inositol polyphosphate phosphatase 1	0.03
Peroxiredoxin-4	0.03
Phospholipase D3	0.03
Procollagen-lysine,2-oxoglutarate 5-dioxygenase 1	0.03
Protein disulfide-isomerase	0.03
Protein FAM188A	0.03
Rho GDP-dissociation inhibitor 1	0.03
RUN domain-containing protein 1	0.03
Thrombospondin-1	0.03
Zinc finger protein 292	0.03
Alpha-N-acetylglucosaminidase	0.02
Angiomotin like 1	0.02
ATP-dependent RNA helicase DDX39A	0.02
Beta-1,3-galactosyl-O-glycosyl-glycoprotein beta-1,6-N-acetylglucosaminyltransferase 3	0.02
Cadherin-3	0.02

CD44 antigen	0.02
Cell division control protein 42 homolog	0.02
Complement component C7	0.02
Dystroglycan	0.02
Endothelin-1	0.02
Gasdermin-A	0.02
Glucosidase 2 subunit beta	0.02
Insulin-like growth factor-binding protein 3	0.02
Laminin subunit beta-2	0.02
Low affinity immunoglobulin epsilon Fc receptor	0.02
Low-density lipoprotein receptor-related protein 11	0.02
Neogenin	0.02
Neutral alpha-glucosidase AB	0.02
Nodal modulator 1	0.02
Plastin-2	0.02
Plexin-B2	0.02
Protein CREG1	0.02
Protein disulfide-isomerase A6	0.02
Protein NDNF	0.02
Ras-related protein Rap-1A	0.02
Receptor-type tyrosine-protein phosphatase F	0.02
RING finger protein 39	0.02
Splicing factor, arginine/serine-rich 15	0.02
Transforming growth factor beta receptor type 3	0.02
Calmodulin	0.01
Collagen alpha-2(IV) chain	0.01
Collagen alpha-3(VI) chain	0.01
Guanine deaminase	0.01
Importin-4	0.01
Macrophage mannose receptor 1	0.01
Nidogen-2	0.01
Protein FAM3C	0.01
IgG FcRN receptor	0.01
Semaphorin-3C	0.01
Transcription factor 25	0.01
von Willebrand factor	0.01

Table S3: Complete list of proteins identified in the late lactation milk proteome

Late Lactation Milk Proteome	% total peptides
Lactotransferrin	49.4
Beta-lactoglobulin	10.53
Ig alpha chain C region	2.16
Immunoglobulin J chain	1.96
Zinc-alpha-2-glycoprotein	1.91
Haptoglobin	1.73
Neutrophil gelatinase-associated lipocalin	1.67
Protein S100-A8	1.67
Lactadherin	1.6
Beta-casein	1.53
Trichosurin	1.44
Polymeric immunoglobulin receptor	1.27
Very early lactation protein	1.25
Hemoglobin subunit alpha	1.07
Sulfhydryl oxidase 1	0.94
Clusterin	0.85
Alpha-1-B glycoprotein-like	0.74
Vimentin	0.74
Alpha-enolase	0.72
Whey acidic protein	0.72
Nucleobindin-1	0.71
Peptidyl-prolyl cis-trans isomerase A	0.71
Transketolase	0.6
Peroxiredoxin-1	0.59
Protein S100-A9	0.59
Ig kappa chain C region	0.58
78 kDa glucose-regulated protein	0.56
Cathepsin L1	0.56
Ig lambda chain V region	0.51
Phosphoglycerate mutase 1	0.51
Actin, cytoplasmic 2	0.5
Alpha-S1-casein	0.47
Ig gamma-1 chain C region	0.46
Kappa Casein	0.45
Alpha-lactalbumin	0.44
Protein disulfide-isomerase A3	0.44
Insulin-like growth factor-binding protein 4	0.42
Envelope glycoprotein (KoRV)	0.4
Ubiquitin-60S ribosomal protein L40	0.4
Pigment epithelium-derived factor	0.39
Profilin-1	0.39
Marsupial Milk 1	0.37
Gelsolin	0.35
Pro-Pol polyprotein (KoRV)	0.3
Retinoic acid receptor responder protein 1	0.3

Xanthine dehydrogenase/oxidase	0.29
Dipeptidase 2	0.27
Ig heavy chain V-III region VH26	0.27
Leukocyte elastase inhibitor	0.27
Glucose-6-phosphate isomerase	0.26
Lysozyme C	0.25
Butyrophilin subfamily 1 member A1	0.22
Parathyroid hormone-related protein	0.22
Extracellular matrix protein 1	0.21
Pyruvate kinase PKM	0.21
Cysteine-rich secretory protein 2	0.2
Leucine-rich alpha-2-glycoprotein	0.2
Beta-2-microglobulin	0.19
Cathepsin F	0.19
Collagen, type VI, alpha 3	0.19
Complement C2	0.19
L-lactate dehydrogenase A chain	0.19
Moesin	0.19
Nucleolar protein of 40 kDa	0.19
Phosphoglycerate kinase 1	0.19
Annexin A1	0.17
Complement C3	0.17
Coronin-1A	0.17
C10orf99-like	0.16
Matrix metalloproteinase-9	0.16
Monocyte differentiation antigen CD14	0.16
Phosphoglucomutase 5	0.16
Thrombospondin-1	0.16
Adenosylhomocysteinase	0.15
Angiopoietin-related protein 4	0.15
Plastin-2	0.15
Transaldolase	0.15
C-C motif chemokine 25	0.14
Cystatin-M	0.14
Resistin-like beta	0.14
Sphingomyelin phosphodiesterase	0.14
Glyceraldehyde-3-phosphate dehydrogenase	0.13
Heat shock cognate 71 kDa protein	0.13
Histone-lysine N-methyltransferase 2C	0.13
Proteasome subunit beta type-2	0.13
L-lactate dehydrogenase B chain	0.12
Peroxiredoxin-5, mitochondrial	0.12
Augurin	0.11
Copper transport protein ATOX1	0.11
Myosin light polypeptide 6	0.11
Pancreatic secretory granule membrane major glycoprotein GP2	0.11
Peptidoglycan recognition protein 1	0.11
Triosephosphate isomerase	0.11
Cathelicidin Phci6	0.1
Cell death activator CIDE-A	0.1

Cytochrome b-245 light chain	0.1
Heat shock protein HSP 90-alpha	0.1
Heat shock protein HSP 90-beta	0.1
Neutral alpha-glucosidase AB	0.1
Adenylyl cyclase-associated protein 1	0.09
Alpha-1-antiproteinase	0.09
Brain acid soluble protein 1 homolog	0.09
Endoplasmic reticulum resident protein 44	0.09
Fructose-bisphosphate aldolase A	0.09
Histone H1.2	0.09
40S ribosomal protein SA	0.08
Glucosidase 2 subunit beta	0.08
Insulin-like growth factor-binding protein 6	0.08
Nucleoside diphosphate kinase B	0.08
Protein DJ-1	0.08
Thymidine phosphorylase	0.08
WAP four-disulfide core domain protein 2	0.08
1,5-anhydro-D-fructose reductase	0.07
Cathelicidin Phci1	0.07
Chloride intracellular channel protein 1	0.07
F-actin-capping protein subunit alpha-1	0.07
Golgi membrane protein 1	0.07
Proteasome subunit alpha type-1	0.07
Transforming growth factor beta receptor type 3	0.07
26S proteasome non-ATPase regulatory subunit 7	0.06
Apolipoprotein E	0.06
Cadherin-1	0.06
Cystatin-C	0.06
Ezrin	0.06
Growth-regulated alpha protein	0.06
High mobility group protein B2	0.06
Homeobox protein Hox-B2	0.06
Hypoxia up-regulated protein 1	0.06
Lamin-B2	0.06
Protein disulfide-isomerase	0.06
Complement C4-A	0.05
Ephrin-A1	0.05
Glutathione reductase, mitochondrial	0.05
Histone H1x	0.05
Hsp90 co-chaperone Cdc37	0.05
Inosine-5'-monophosphate dehydrogenase 2	0.05
Lipoprotein lipase	0.05
Macrophage-capping protein	0.05
Nucleobindin-2	0.05
Programmed cell death 6-interacting protein	0.05
Protein FAM20A	0.05
Protein kinase C and casein kinase substrate in neurons protein 2	0.05
Tigger transposable element-derived protein 5	0.05
Vasodilator-stimulated phosphoprotein	0.05
14-3-3 protein beta/alpha	0.04

Actin-related protein 2/3 complex subunit 1B	0.04
Alpha-2-macroglobulin	0.04
Alpha-actinin-1	0.04
ATP synthase subunit beta, mitochondrial	0.04
Carboxypeptidase D	0.04
Catalase	0.04
Cytosol aminopeptidase	0.04
Glia-derived nexin	0.04
Granulins	0.04
Heterogeneous nuclear ribonucleoprotein D0	0.04
Lysosome-associated membrane glycoprotein 1	0.04
Malate dehydrogenase, mitochondrial	0.04
Plasminogen activator inhibitor 1 RNA-binding protein	0.04
Protein disulfide-isomerase A6	0.04
Protein OS-9	0.04
Protein-L-isoaspartate(D-aspartate) O-methyltransferase	0.04
Ras-related protein Rap-1b	0.04
Ribonuclease 8	0.04
Transforming protein RhoA	0.04
Tropomyosin alpha-3 chain	0.04
Unknown	0.04
10 kDa heat shock protein, mitochondrial	0.03
Acid sphingomyelinase-like phosphodiesterase 3a	0.03
Actin-related protein 3	0.03
Calsyntenin-1	0.03
Calumenin	0.03
Cytoskeleton-associated protein 4	0.03
Endoplasmin	0.03
Fibronectin	0.03
Gag polyprotein (KoRV)	0.03
Histone H2B type 2-E	0.03
Kynurenine--oxoglutarate transaminase 3	0.03
Leukotriene A-4 hydrolase	0.03
Perilipin-2	0.03
Platelet-derived growth factor subunit A	0.03
Procollagen-lysine,2-oxoglutarate 5-dioxygenase 3	0.03
Prosaposin	0.03
Protein NOXP20	0.03
Protein phosphatase 1 regulatory subunit 26	0.03
Src substrate cortactin	0.03
Succinyl-CoA ligase [ADP-forming] subunit beta, mitochondrial	0.03
Talin-1	0.03
Transforming growth factor-beta-induced protein ig-h3	0.03
Unconventional myosin-Id	0.03
1,4-alpha-glucan-branching enzyme	0.02
Aconitate hydratase, mitochondrial	0.02
Beta-1,4-galactosyltransferase 1	0.02
Beta-galactoside alpha-2,6-sialyltransferase 1	0.02
Calcium-independent phospholipase A2-gamma	0.02
CapZ-interacting protein	0.02

CD44 antigen	0.02
Cell division control protein 42 homolog	0.02
Coatomer subunit delta	0.02
Complement C1r subcomponent	0.02
Cyclic AMP-responsive element-binding protein 3-like protein 2	0.02
Eukaryotic translation initiation factor 5A-2	0.02
Exostosin-1	0.02
Extracellular serine/threonine protein kinase FAM20C	0.02
Family With Sequence Similarity 168, Member B	0.02
G-protein coupled receptor 56	0.02
Laminin subunit beta-2	0.02
Low-density lipoprotein receptor-related protein 11	0.02
Lysosomal alpha-mannosidase	0.02
Myotubularin-related protein 6	0.02
Platelet glycoprotein 4	0.02
Protein BEAN1	0.02
Puromycin-sensitive aminopeptidase	0.02
Pyridoxal kinase	0.02
Ras GTPase-activating-like protein IQGAP1	0.02
Receptor-type tyrosine-protein phosphatase F	0.02
RecQ-mediated genome instability protein 1	0.02
Serine/threonine-protein kinase 17B	0.02
Sperm-associated antigen 1	0.02
Splicing factor, arginine/serine-rich 15	0.02
Translation factor GUF1, mitochondrial	0.02
Tubulin beta-7 chain	0.02
Ubiquitin-conjugating enzyme E2 J1	0.02
Collagen alpha-2(IV) chain	0.01
Eukaryotic translation initiation factor 4H	0.01
Golgi apparatus protein 1	0.01
Insulin-like growth factor I	0.01
Insulin-like growth factor-binding protein 5	0.01
Interleukin enhancer-binding factor 3	0.01
Nidogen-1	0.01
Pantothenate kinase 3	0.01
Protein FAM208B	0.01
Sine oculis-binding protein homolog	0.01
Structural maintenance of chromosomes protein 1A	0.01
UDP-glucose:glycoprotein glucosyltransferase 1	0.01
Vacuolar protein sorting-associated protein 54	0.01

Table S4: List of immune transcripts identified in mammary gland transcriptome

Gene	% total
FTH1	1.5542
BTN1A1	0.6011
AZGP1	0.4279
MHCI	0.1966
FTL	0.1418
TPT1	0.1150
MUC1	0.1054
CD74	0.1010
B2M	0.0962
CD63	0.0960
PIGR	0.0904
MHCI	0.0827
CCL25	0.0801
CD9	0.0775
PRDX1	0.0753
Phci7	0.0669
MHCI	0.0642
C1QA	0.0641
MHCII-DAB	0.0580
MHCII-DAA	0.0525
C1S	0.0517
CTGF	0.0495
Phci1	0.0491
IFITM2	0.0440
SERPING1	0.0389
THBS1	0.0374
HSPA1L	0.0359
C1QC	0.0355
ANXA5	0.0348
CALR	0.0347
EPCAM	0.0338
CLEC3B	0.0337
CCLK4	0.0330
CD47	0.0322
ACKR4	0.0313
APLP2	0.0302
BCAP31	0.0302
MIF	0.0293
HM13	0.0290
PRDX2	0.0274
ITGB1	0.0272
LGALS1	0.0271
DAP1	0.0268
THY1	0.0254
PSMD8	0.0244

FCGR2	0.0240
MME	0.0233
IL25	0.0230
IL1RL2	0.0228
IL10RB	0.0219
JUN	0.0216
MHCII	0.0213
LRPAP1	0.0197
VTCN1	0.0197
PDIA3	0.0191
CASP14	0.0190
FKBP1A	0.0189
CTSL	0.0187
PDCD4	0.0186
PBX1	0.0184
ISLR	0.0184
S10A2	0.0183
CXCL12	0.0181
PRNP	0.0180
HPT	0.0179
AIMP1	0.0178
IL20RA	0.0177
PPIB	0.0172
GADD45A	0.0165
BTN2A2	0.0159
EDN1	0.0159
PSMB7	0.0157
MAGEC1	0.0155
PSMD2	0.0155
MCAM	0.0155
PSMB2	0.0153
HSPD1	0.0152
PSMB1	0.0147
BSG	0.0147
PSMC4	0.0144
TUBB5	0.0144
CD14	0.0143
TSC22D3	0.0142
CANX	0.0141
F11R	0.0139
PSME2	0.0138
LTF	0.0138
CCL20	0.0138
ANXA11	0.0136
LYZ	0.0135
CD164	0.0135
MAPK3	0.0134
LAP3	0.0132
CD36	0.0131
BANF1	0.0128

STAT3	0.0128
MHCI	0.0128
CCL21	0.0124
CSNK2	0.0123
JUND	0.0122
CO4A	0.0122
SLC11A1	0.0120
IFI30	0.0119
STAT2	0.0119
ARHGDIG	0.0119
PVRL2	0.0119
FGL2	0.0119
BRD2	0.0118
FKBP8	0.0114
PSMD7	0.0111
JAK1	0.0111
RELA	0.0110
PSME1	0.0109
MHCII	0.0106
VATG1	0.0106
ARF6	0.0104
PSMA6	0.0102
FCER1G	0.0102
PACS1	0.0102
IK	0.0101
STAT5A	0.0101
PSA1	0.0101
AGPAT1	0.0101
NCOA4	0.0101
XIAP	0.0100
PGF	0.0100
SOD2	0.0098
IER3	0.0097
CTSH	0.0096
CEBPB	0.0096
ITGA6	0.0093
SLC3A2	0.0092
BRD2	0.0092
TAP1	0.0091
MAPK1	0.0091
LIPL	0.0091
IL1R2	0.0090
ENG	0.0090
LY96	0.0090
C2	0.0089
ADIPOQ	0.0089
TYROBP	0.0088
WARS	0.0087
CCL19	0.0087
CFLAR	0.0086

BAX	0.0086
LITAF	0.0085
BAT1	0.0085
IL4RA	0.0085
HMGB2	0.0084
ASC	0.0084
AXL	0.0083
ITGA2	0.0083
PSMA4	0.0082
TNFRSF1A	0.0082
PSB3	0.0081
DDAH2	0.0081
A2MG	0.0081
ADAM9	0.0081
SET	0.0079
F10	0.0079
TNFSF10	0.0079
ERBB3	0.0078
IL13RA1	0.0077
DDT	0.0077
XBP1	0.0076
BCAM	0.0076
TBB2A	0.0075
INGR1	0.0074
SIRPA	0.0074
IFNAR1	0.0074
JUNB	0.0073
C1R	0.0072
TAPBP	0.0072
PDGFRB	0.0070
MCFD2	0.0070
CTL2	0.0069
MHCI	0.0069
AIF1	0.0069
MHCI	0.0069
PSA7	0.0068
CTSS	0.0067
PLSCR4	0.0067
SCARB1	0.0067
NFKBIA	0.0067
ILF2	0.0066
ATF6B	0.0066
VWF	0.0066
OAS1	0.0065
NT5E	0.0065
TNR3	0.0065
SEM3C	0.0065
BAT5	0.0065
PSA2	0.0065
CD44	0.0065

FN1	0.0064
RAF1	0.0064
PDIA4	0.0064
MAX	0.0064
RN112	0.0064
DMA	0.0064
GADD45B	0.0064
ACKR3	0.0063
PSMD13	0.0063
C1QBP	0.0063
SRGEF	0.0063
LAMB1	0.0062
PDGFA	0.0062
PBX2	0.0062
IL6ST	0.0061
NRP1	0.0061
CCLK5	0.0061
BAG6	0.0061
SIVA	0.0060
PA1B3	0.0058
CADH5	0.0058
MAPK14	0.0058
CD46	0.0058
EIF6	0.0057
EFEMP2	0.0057
PTGES	0.0056
SART3	0.0056
ADML	0.0056
SIRPB1	0.0056
TAP2B	0.0056
MAPK1	0.0056
ITGB1	0.0055
CASP3	0.0054
CASP8	0.0054
VDR	0.0054
TFR1	0.0054
HIMAP4	0.0054
CAMLG	0.0054
LSM2	0.0053
PDCD6	0.0053
PRRC2A	0.0053
MLLT4	0.0052
NINJ1	0.0052
ADAM17	0.0052
TMX1	0.0052
S100A9	0.0052
FCER2	0.0052
S100A8	0.0052
ERAP1	0.0051
SEM3A	0.0051

IL1RN	0.0050
MAP4K4	0.0050
PSMA5	0.0050
SERPINB9	0.0050
KIT	0.0049
KBRS2	0.0049
PSMD1	0.0049
AHR	0.0049
CCLK3	0.0048
BAG3	0.0048
ARNT	0.0048
MRC1	0.0048
EHMT2	0.0048
PRDX3	0.0048
ABI1	0.0048
ISGF3G	0.0047
SCAR3	0.0047
PAR3	0.0047
CD53	0.0047
MAP3K1	0.0047
IKBKG	0.0047
CFAB	0.0046
SFTPD	0.0046
ELMO3	0.0046
MAFB	0.0046
ETS1	0.0045
TNFAIP1	0.0045
F2RL1	0.0044
KIG8	0.0044
IRF2	0.0044
TLR2	0.0044
GATA3	0.0044
GGCX	0.0044
MP2K1	0.0043
PSMA3	0.0043
TOLIP	0.0043
IGHK	0.0043
SIGIRR	0.0043
AOX1	0.0043
ILF3	0.0043
PTGS2	0.0042
SOCS2	0.0042
RNF5	0.0041
SEM7A	0.0040
LGALS9	0.0040
LMAN1	0.0040
IRF6	0.0040
SIGLEC9	0.0040
ACP1	0.0040
IGSF8	0.0039

SOCS3	0.0039
NFKB2	0.0039
ARHGDIB	0.0039
STAT5B	0.0038
PDGFB	0.0038
PRKCD	0.0038
PSD11	0.0038
BIRC2	0.0038
B3GALT3	0.0037
HM13	0.0037
F8	0.0037
TNFSF10L	0.0036
PLXB2	0.0036
NKRF	0.0035
CNIH1	0.0035
PSMD12	0.0035
SAMHD1	0.0035
IL11RA	0.0034
LBP	0.0034
IMA5	0.0034
CCLK2	0.0034
PSMD9	0.0034
YARS	0.0034
UBE2H	0.0034
SPG21	0.0034
NCR2	0.0034
CTF1	0.0033
PSMC2	0.0033
CSK	0.0033
TRIM14	0.0033
TNFRSF11A	0.0033
DCBLD2	0.0033
MAEA	0.0033
PSMB8	0.0033
B2L10	0.0033
F8A3	0.0033
WASL	0.0033
ACVR1B	0.0033
IL8	0.0032
MAPK8	0.0032
RNASE7	0.0032
CISH	0.0032
CBL	0.0032
BCL7C	0.0032
TSTA3	0.0032
ABCE1	0.0032
PSMD4	0.0031
AGER	0.0031
CD68	0.0031
ADAR	0.0031

F3	0.0031
TFPI	0.0031
FCN1	0.0031
SP2	0.0031
ACVR1	0.0031
ITGAV	0.0031
NR3C1	0.0031
MAP4K2	0.0030
HNMT	0.0030
ICAM1	0.0030
CHUK	0.0030
CD276	0.0030
TSN8	0.0030
ID1	0.0029
LY6G6D	0.0029
ZBTB12	0.0029
MAD4	0.0029
VEGFB	0.0028
SIGMAR1	0.0028
PROS1	0.0028
PVRL1	0.0028
TF7L1	0.0028
IL1RAP	0.0028
CFAH	0.0028
CMKLR1	0.0028
RAC3	0.0028
TLR4	0.0028
TMED1	0.0028
ALOX5AP	0.0028
CD2AP	0.0028
VPP1	0.0027
IRF3	0.0027
STK19	0.0027
ABCA1	0.0027
ATF1	0.0027
CF047	0.0027
LRP1	0.0027
PTN6	0.0026
VEGFA	0.0026
LGALS8	0.0026
DDA1	0.0026
PML	0.0026
PTK2	0.0026
IFIT5	0.0026
WIPF1	0.0026
FOXK2	0.0026
MAP3K3	0.0025
ITAX	0.0025
MYD88	0.0025
DXO	0.0025

CXCL1LA	0.0025
ATL2	0.0025
IFIT1	0.0025
PRKRIR	0.0025
TAPBPL	0.0025
KIG18	0.0025
MMD	0.0025
ASC	0.0024
SFSWAP	0.0024
GTPB1	0.0024
RETN	0.0024
OASL2	0.0024
COLEC12	0.0024
GBP1	0.0024
F13A1	0.0023
ABCF1	0.0023
TCF4	0.0023
TNFAIP6	0.0023
NFIL3	0.0023
B2CL2	0.0023
TRAF4	0.0023
ELK4	0.0023
SKIV2L	0.0023
TNFSF12	0.0023
ORM1	0.0023
CASP6	0.0023
DOCK1	0.0022
NCOA6	0.0022
CD34	0.0022
MAPK2	0.0022
BCL3	0.0022
KBRAS1	0.0022
NEUR1	0.0022
CATH	0.0022
ALCAM	0.0022
TRAF2	0.0022
MLLT6	0.0022
MHCII	0.0022
MLLT1	0.0022
CXCR4	0.0022
RIPK1	0.0022
DAXX	0.0022
RAC2	0.0021
C4B	0.0021
IL17RC	0.0021
PPP1R10	0.0021
SIAT1	0.0021
PTGER4	0.0021
EGR1	0.0021
NOTCG4	0.0021

PSMD5	0.0021
MRC2	0.0021
I17RA	0.0020
EHMT1	0.0020
PTN9	0.0020
NFIB	0.0020
SPA17	0.0020
PSMF1	0.0020
M4K3	0.0020
NFATC3	0.0020
ATP6V0A2	0.0020
ILF3	0.0020
C43BP	0.0019
PLAU	0.0019
IRAK4	0.0019
CLC4G	0.0019
VARS	0.0019
IRF1	0.0019
PADI1	0.0019
AIF1L	0.0019
VPP3	0.0019
GIMA5	0.0019
KANK1	0.0019
B3GNT6	0.0019
TP4AP	0.0019
PTPN1	0.0019
LRBA	0.0019
MPL	0.0019
ALOX5	0.0018
TSPAN7	0.0018
TNAP2	0.0018
PGLYRP2	0.0018
SYTL1	0.0018
FKBP5	0.0018
LPAR1	0.0018
CD37	0.0018
CCR5	0.0018
C1QTNF5	0.0018
FADD	0.0018
AATK	0.0017
CXL14	0.0017
TGFB2	0.0017
IKBKAP	0.0017
BRD3	0.0017
TAB2	0.0017
BAD	0.0017
DDR2	0.0017
CFAD	0.0017
IGF2R	0.0017
PTN13	0.0017

LIF	0.0017
DIDO1	0.0017
CD300A	0.0016
GBP6	0.0016
ATRN	0.0016
SOCS1	0.0016
CEBPB	0.0016
IRAK2	0.0016
HSF1	0.0016
ITGA1	0.0016
CD82	0.0016
PTX3	0.0016
OSMR	0.0016
TNFRSF6	0.0016
TNFRSF1B	0.0016
MAD1	0.0016
ITGAL	0.0015
ABL1	0.0015
CXCL10A	0.0015
CATHb (CATH3)	0.0015
C4BPA	0.0015
DAP3	0.0015
BAK1	0.0015
ARRB2	0.0015
CXCL13	0.0015
BAT4	0.0015
CCND1	0.0015
KITLG	0.0015
PROCR	0.0015
CYTH1	0.0015
MED1	0.0015
TANK	0.0015
IGHG	0.0014
KIG7	0.0014
BCL10	0.0014
FGFP2	0.0014
CSF1	0.0014
KNG1	0.0014
GYPC	0.0014
C7	0.0014
BAG4	0.0014
CEACAM1	0.0013
PRC2B	0.0013
PNCK	0.0013
ADK	0.0013
BAG1	0.0013
SERPINE1	0.0013
IL33	0.0013
FTH1	0.0013
KMT2A	0.0013

PVRL3	0.0013
GCNT2	0.0013
TNFRSF21	0.0012
NFATC3	0.0012
SOCS6	0.0012
ARRB1	0.0012
C5AR1	0.0012
PCGF2	0.0012
MAF	0.0012
SMARCAD1	0.0012
CBLC	0.0012
HM13	0.0012
MAP4K1	0.0012
ITCH	0.0012
APLP1	0.0012
TREM2	0.0012
ENTPD1	0.0012
CCLK6	0.0012
GNAQ	0.0012
TGFB3	0.0012
CLC4K	0.0011
NOD1	0.0011
IFIT5	0.0011
PSB9	0.0011
EDA2R	0.0011
IRF5	0.0011
TNFSF13L	0.0011
TNXB	0.0011
IL6RA	0.0011
TNC	0.0011
GNAQ	0.0011
CASP10	0.0011
APLN	0.0011
ELMO2	0.0011
HCK	0.0011
PTK2B	0.0011
MAP2K7	0.0011
MCM3AP	0.0011
CD200R	0.0011
FCGR3	0.0011
IGK	0.0011
NCF2	0.0011
NFKBIZ	0.0011
GSTM5	0.0011
TNFRSF14	0.0011
IL31R	0.0011
CCLK1	0.0011
TLR1	0.0011
PPARD	0.0011
TGIF2	0.0011

ATM	0.0010
PPT2	0.0010
VEGFC	0.0010
TGFA	0.0010
RFXANK	0.0010
ABCA7	0.0010
MAP3K7	0.0010
TNFRSF10C	0.0010
FOXN2	0.0010
SERPINA3	0.0010
IGSF3	0.0010
CBP	0.0010
NOTCH3	0.0010
HSF2	0.0010
ELMO1	0.0010
FUT1	0.0010
IL1R1	0.0010
CD109	0.0010
WAS	0.0010
LCP2	0.0010
IGJ	0.0010
KIG13	0.0010
ATP6V1G2	0.0010
PACS2	0.0009
CBLB	0.0009
NOTCH1	0.0009
SLC7A5	0.0009
LYAM1	0.0009
BC11A	0.0009
OASL2	0.0009
AA1R	0.0009
TNFAIP3	0.0009
CX3CR1	0.0009
RFXAP	0.0009
SPTB1	0.0009
OAS3	0.0009
RIPK2	0.0009
IKBL1	0.0009
SIGLEC	0.0009
IL3RA or CSF2RA	0.0009
CCND3	0.0009
IL18	0.0009
TNFRSF6B	0.0009
IL36L1	0.0009
MARCH8	0.0009
CD5L	0.0009
AFF1	0.0009
CCND2	0.0009
IKBP1	0.0008
CCR1	0.0008

TRAF6	0.0008
JAM2	0.0008
MAP2K4	0.0008
EMR1	0.0008
IKZF5	0.0008
IL5	0.0008
ITGA3	0.0008
KPTN	0.0008
CSF2RB	0.0008
GNE	0.0008
NFATC1	0.0008
PREX1	0.0008
GPR183	0.0008
EBF1	0.0008
CLEC4F	0.0008
TXLNA	0.0008
CCL28	0.0008
ERBB2	0.0008
GPSM3	0.0008
CLECSF6	0.0008
TNFSF0	0.0008
C1R	0.0008
ISG15	0.0008
BCL7B	0.0007
PABP4	0.0007
IRF8	0.0007
NCF1	0.0007
FKBPL	0.0007
INPP5D	0.0007
MAP3K4	0.0007
ATRN	0.0007
IL36RN	0.0007
CD84	0.0007
CBFA2T2H	0.0007
RFX1	0.0007
PAFAH2	0.0007
ENTPD5	0.0007
CYP2U1	0.0007
TNFRSF18	0.0007
MAPK13	0.0007
C1QT2	0.0007
PLA2G4A	0.0007
BKRB2	0.0007
MLLT10	0.0007
NFAT5	0.0007
PADI4	0.0007
VCAM1	0.0007
ICOSL	0.0007
CCRL2	0.0007
TLR8	0.0007

TNFSF7	0.0006
VARS2	0.0006
CREBBP	0.0006
CYSLTR1	0.0006
BTK	0.0006
CP	0.0006
TDP2	0.0006
GAB2	0.0006
TBXAS1	0.0006
CASP4	0.0006
IRAK1	0.0006
SEMA4D	0.0006
PPARG	0.0006
DST	0.0006
CX3CL1	0.0006
NKTR	0.0006
TXLNG	0.0006
DAPK1	0.0006
IGHA	0.0006
SLAMF9	0.0006
LY86	0.0006
SCARF1	0.0006
C1QT1	0.0006
HTR6	0.0006
SRC	0.0006
IL10RA	0.0006
CLEC11A	0.0006
ZEB1	0.0006
SREC2	0.0006
CASP2	0.0006
CRISP3	0.0006
IFIT3	0.0006
CSF3R	0.0006
BIRC5	0.0006
PVRL2	0.0006
TLR5	0.0006
BCL2	0.0006
IGHM	0.0005
MAPK5	0.0005
CLEC1A	0.0005
GEM	0.0005
MHCI	0.0005
PRKR	0.0005
IFNGR2	0.0005
LGALS3	0.0005
SN	0.0005
RN216	0.0005
PDL2	0.0005
DGKD	0.0005
SPP1	0.0005

CCL24	0.0005
TLR1	0.0005
CHST4	0.0005
INDO1	0.0005
HAS2	0.0005
CXCL5L	0.0005
MAP3K2	0.0005
ICAM2	0.0005
DARC	0.0005
HMHA1	0.0005
HBEGF	0.0005
LRC1	0.0005
C8AP2	0.0005
IL17B	0.0005
PDE7A	0.0005
CYP2C19	0.0005
IRAK3	0.0005
CREM	0.0005
DEFB19	0.0004
DPP8	0.0004
FKBP1B	0.0004
PDCD7	0.0004
CLEC4E	0.0004
MAP3K14	0.0004
PSMB10	0.0004
C1QTNF6	0.0004
MOG	0.0004
TCAM1	0.0004
TLR7	0.0004
PTGIR	0.0004
PTPRC	0.0004
MMP9	0.0004
DEF6	0.0004
EDA	0.0004
VS10L	0.0004
PLXA1	0.0004
ITA7	0.0004
TNFRSF5	0.0004
TRIM32	0.0004
VIPR1	0.0004
CD226	0.0004
LYZ	0.0004
CP2K1	0.0004
MUC13	0.0004
IGL	0.0004
CCR6	0.0003
TAL1	0.0003
MHC2TA	0.0003
KCC1A	0.0003
IL17D	0.0003

IL21R	0.0003
FLT3	0.0003
SEM3G	0.0003
MVK	0.0003
IL7RA	0.0003
IL16	0.0003
IRF4	0.0003
PIGA	0.0003
MLLT3	0.0003
BKRB1	0.0003
FAIM3	0.0003
AFF3	0.0003
VAV1	0.0003
FCRLA	0.0003
DOCK2	0.0003
I17RE	0.0003
ICOS	0.0003
AIM2	0.0003
PGRP1	0.0003
TNFRSF10B	0.0003
DAF	0.0003
KIG3	0.0003
VAV3	0.0003
IGHL	0.0003
PTGS1	0.0003
CP1A1	0.0003
IGK	0.0003
PTAFR	0.0003
CLEC5A	0.0003
MOG	0.0003
CSF2RA	0.0003
BC11A	0.0003
ABCG4	0.0003
NRP2	0.0003
IKZF2	0.0003
ATL1	0.0003
TNFRSF19	0.0003
IKBKE	0.0003
MHCI	0.0003
IKZF4	0.0003
AVPR1A	0.0003
MAP3K8	0.0003
RELB	0.0002
KIG10	0.0002
ADAM9	0.0002
TICAM2	0.0002
LT4R2	0.0002
TNFSF8	0.0002
NFATC2	0.0002
IGHV	0.0002

TRAF3	0.0002
PDCD1LG1	0.0002
TRAC	0.0002
TNFSF13B	0.0002
SLC11A1	0.0002
BLNK	0.0002
